# Emergency Preparedness and Management of Mobile Cabin Hospitals in China During the COVID-19 Pandemic

**DOI:** 10.3389/fpubh.2021.763723

**Published:** 2022-01-03

**Authors:** Fang Shi, Hao Li, Rui Liu, Yan Liu, Xiaoxue Liu, Haoyu Wen, Chuanhua Yu

**Affiliations:** ^1^Department of Epidemiology and Biostatistics, School of Public Health, Wuhan University, Wuhan, China; ^2^Global Health Institute, Wuhan University, Wuhan, China; ^3^National Health Commission Key Lab of Radiation Biology, Jilin University, Changchun, China

**Keywords:** mobile cabin hospital, Fangcang shelter hospital, management, COVID-19, China

## Abstract

The healthcare systems in China and globally have faced serious challenges during the coronavirus disease (COVID-19) pandemic. The shortage of beds in traditional hospitals has exacerbated the threat of COVID-19. To increase the number of available beds, China implemented a special public health measure of opening mobile cabin hospitals. Mobile cabin hospitals, also called Fangcang shelter hospitals, refer to large-scale public venues such as indoor stadiums and exhibition centers converted to temporary hospitals. This study is a mini review of the practice of mobile cabin hospitals in China. The first part is regarding emergency preparedness, including site selection, conversion, layout, and zoning before opening the hospital, and the second is on hospital management, including organization management, management of nosocomial infections, information technology support, and material supply. This review provides some practical recommendations for countries that need mobile cabin hospitals to relieve the pressure of the pandemic on the healthcare systems.

## Introduction

The coronavirus disease (COVID-19) pandemic has become a health catastrophe, and healthcare systems worldwide have been overwhelmed by the new surge of infections (1). The substantial medical needs of the large number of COVID-19 patients are straining the healthcare system in India, where the wards are limited (2). Dr. Anthony Fauci, a top pandemic expert and the chief medical advisor of the U.S. government, made the following recommendations to control the pandemic in India: establishing lockdown for a couple of weeks, setting up emergency units as hospitals as done in China, and having a central organization (3). The emergency units mentioned refer to China's mobile cabin hospitals.

Mobile cabin hospital, also called Fangcang shelter hospital, is a type of modular health equipment providing multiple functions, such as isolation, triage, basic medical care, frequent monitoring, rapid referral, and essential living needs (4). Mobile cabin hospitals help solve the issues of bed shortages and separate mild cases from serious ones (5). According to the clinical manifestations, COVID-19 cases can be divided into mild, moderate, severe, and critical types (6). Approximately 80% of the COVID-19 cases are of the mild or moderate types that do not require intensive care, and these patients are able to walk around by themselves. Without centralized management, the infection can spread rapidly in the community (7, 8). In the early stages of the epidemic, medical facilities were insufficient (9). To ensure early isolation and treatment of mild and moderate cases, the Chinese government designed and built mobile cabin hospitals (10). Mobile cabin hospitals provided a large number of beds for mild and moderate COVID-19 cases, excluding the elderly, pregnant women, and those with pre-existing health conditions. This changed the family-based quarantine approach into group isolation of mild cases, obviating within-household and community transmissions (11–13).

The World Health Organization has recommended “cohort nursing” for large outbreaks, such as influenza (14). Cohort nursing refers to the grouping of patients with the same laboratory-confirmed pathogen in the same isolated area (15). The main difference between cohort nursing and mobile cabin hospitals is that patients are placed in the existing wards in the former, whereas the latter are usually created by converting large-scale public venues such as indoor stadiums, conference centers, or exhibition centers (16). When there are numerous patients and insufficient wards, mobile cabin hospitals can be recommended as an alternative strategy to cohort nursing to control the spread of disease. With the characteristics of rapid construction, massive scale, and low cost, the mobile cabin hospitals can effectively relieve the pressure of the pandemic on healthcare systems (4). For example, the construction of the Hongshan Sports Stadium mobile cabin hospital took only 37 h and admitted over 1,000 patients (17). Since February 5, 2020, 16 mobile cabin hospitals were functional in Wuhan, providing more than 13,000 beds and admitting over 12,000 patients with COVID-19 (16). All patients of the 16 mobile cabin hospitals were discharged by March 10, 2020, and no deaths were reported (18).

**Figure 1:**
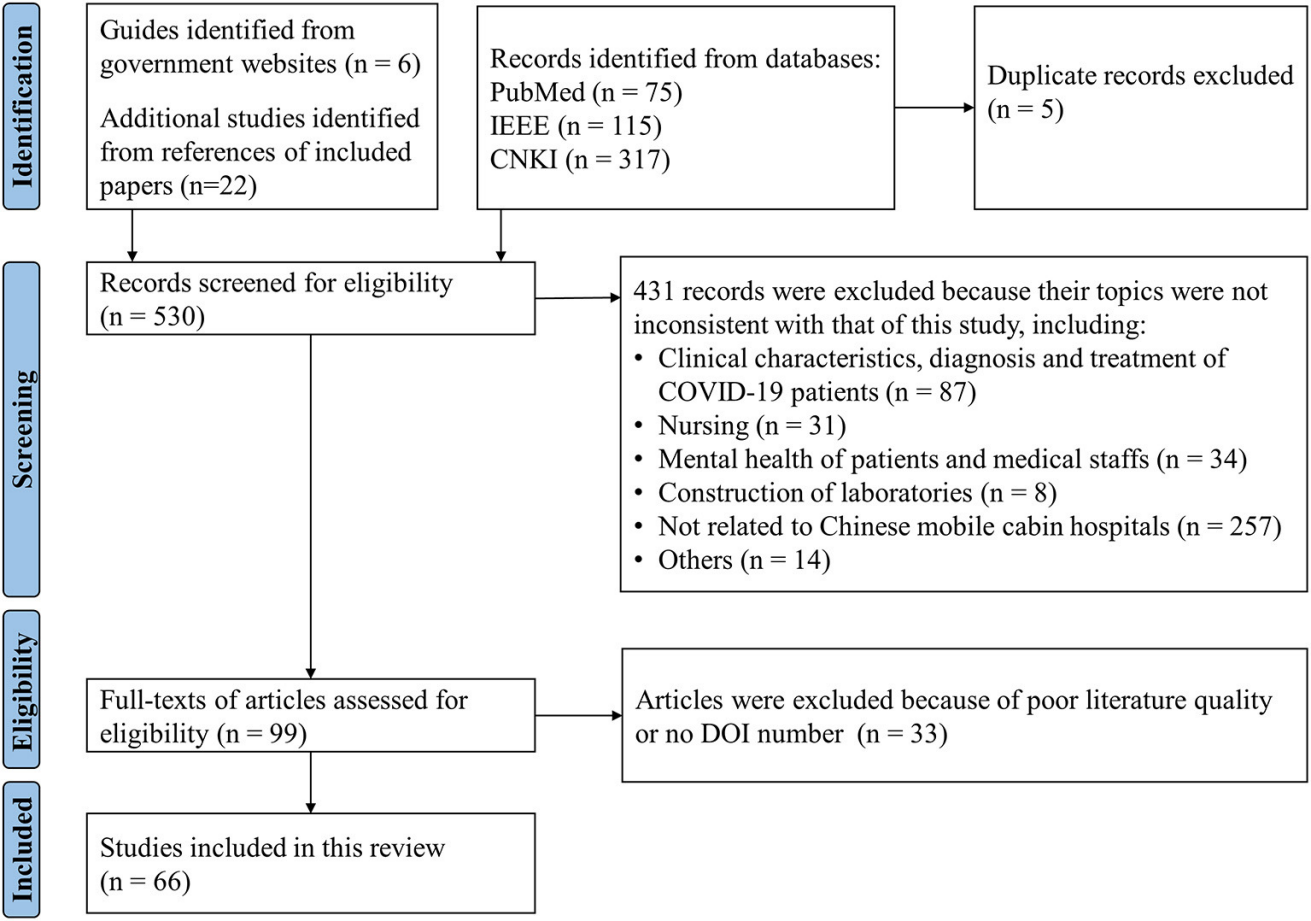
PRISMA flow diagram illustrating the screening process for the literature included in this review.

This article aims to review China's experience in operating mobile cabin hospitals to provide a reference for other countries that can utilize mobile cabin hospitals to relieve the pressure of the COVID-19 pandemic on healthcare systems. The search terms and literature reviewing process were shown in Supplementary Materials and Figure 1. We believe that this experience is valuable even for preparedness against the future outbreak of other respiratory infectious diseases.

## Preparedness of Mobile Cabin Hospitals

### Site Selection

To serve as mobile cabin hospitals, the public buildings should meet the following criteria (10, 19–24):

① Road accessibility, for example, proximity to arteries and main roads;
② Away from the headwaters and densely populated areas;
③ Spacious outdoor area and sufficient indoor space;
④ Presence of electrical, plumbing, and ventilation systems and other infrastructure (buildings with mechanical ventilation systems preferred);
⑤ Easy to remodel, such as interior equipment that can be rapidly removed;
⑥ Fireproof degree and firefighting facilities compliant with fire regulations.

### Conversion of Public Venues Into Hospitals

The design and remodeling of mobile cabin hospitals must meet the standards of infectious disease hospitals (10). Moreover, minimal building intervention is essential to ensure rapid project delivery; hence, the original infrastructure should be fully utilized (25).

① Electricity systems: The transformation of the power system should minimize the impact on the fire protection system of the original site. There should be a reliable high-power electricity supply system. Additionally, a backup power supply system and emergency lighting system are necessary (24). Nursing stations and medical office areas should be equipped with a certain number of sockets to facilitate the routine work of the medical staff. Furthermore, the sockets provided in the hospital bed area should meet the needs of patients to use low-power electrical equipment, such as mobile phones and lamps (21).

② Ventilation: The airflow should be blown from the clean area to the contaminated area (23); therefore, it is recommended to set up a mechanical ventilation system to control the airflow in the hospital (19). Besides, air purifiers can be used in contaminated and semi-contaminated zones to reduce the possible virus-laden aerosols (26).

③ Toilets: Medical staff and patients' toilets should be kept separate, and foam-blocked mobile toilets are preferred (22). Toilets should be built downwind, away from the dining areas and water points. All toilet feces must be strictly disinfected and subjected to concentrated harmless treatment under the requirements of the infectious disease hospital, wherein direct discharge is strictly prohibited (27).

④ Water supply: The centralized water supply system should be equipped with sterilization and disinfection facilities (28). Furthermore, the pumping house and hot water room should be set in a clean area. Each nursing group should set up a water supply point, and drinking water points for medical staff and patients should be kept separate (23, 29).

⑤ Sewage: Sewage from mobile cabin hospitals, including condensate water from air conditioners, should not be directly discharged, and temporary tanks for sewage treatment should be established (30). Additionally, an automatic monitoring system for water quality should be installed at treated sewage outlets to ensure that the discharged sewage meets the standards (31).

⑥ Fire protection: It is necessary to ensure that the automatic fire alarm system and fire facilities of the original building can be used normally (23). There should be at least two safety exits in different directions in the ward (19). Additionally, each medical staff member should be equipped with a firefighting self-rescue respirator (22).

⑦ Heating or cooling: It is not recommended to use centralized air conditioning to adjust the indoor temperature in order to prevent cross-infection. While split air conditioning can be used to cool down in summer, electric heating blankets and electric oil heaters can be used for heating in winter (19). Additionally, mobile cabin hospitals in Wuhan prepared quilts and down coats for each patient.

### Architectural Layout

The layout of a mobile cabin hospital follows the standards of infectious disease hospitals, which are partitioned into three zones (contamination zone, semi-contamination zone, and clean zone) and two passages (staff passage and patient passage) (4). The contaminated zone refers to areas where patients reside and receive treatment, comprising the wards, treatment rooms, waste rooms, and places of activity. The clean zone includes the medical staff's dressing room, catering room, duty room, and warehouse. The semi-contaminated zone is the area between the clean and contaminated zones, comprising the medical staff's offices, nurse stations, medical equipment areas, and other areas that may be contaminated by patients (10). Each zone should be marked and isolated, and fixed routes must be set for the medical staff to enter and leave the contaminated zone (22). Partition materials shall be anti-inflammable with height of at least 1.8 meters (21, 24).

### Functional Zoning

According to the medical functions, mobile cabin hospitals are mainly divided into the following sections (32, 33):

① Ward: It is the core area of a mobile cabin hospital, which is divided into different sections for male and female patients and further divided into the general area and key observation area according to whether the patients have underlying diseases. In the patient ward, beds are at least 1.2 m away from each other and equipped with hand sanitizer at the end of each bed (5). In case of double-row beds, a distance of at least 1.4 m is maintained between the ends of close beds (19, 21).

② Image testing area: This area is composed of multiple sets of imaging examination vehicles and various imaging techniques such as radiography, computed tomography, and ultrasonography are conducted.

③ Routine laboratory testing area: Routine laboratory inspection tasks, such as routine blood testing of patients, are performed in this area.

④ Virus nucleic acid detection area: It is composed of mobile P3 laboratories (34).

⑤ Intensive observation and treatment area: This area is equipped with oxygen cylinders, rescue medicines, and monitoring equipment to provide treatment and nursing for patients whose conditions worsen during hospitalization (22).

## Management of Mobile Cabin Hospitals

### Organizational Management

**Figure 2:**
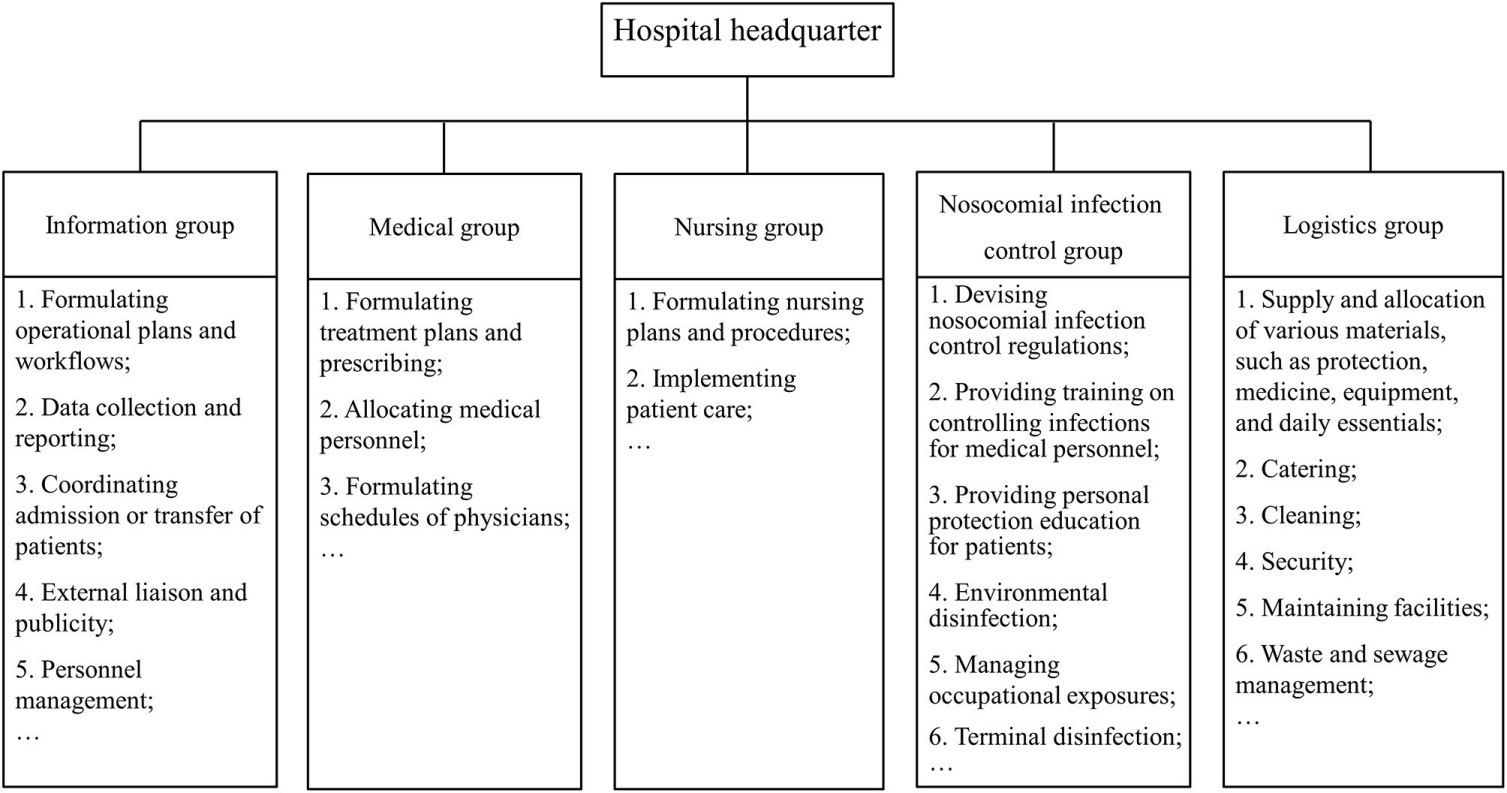
Organizational structure of the mobile cabin hospital.

Clarifying each person's responsibilities and ensuring the stability of the management team are prerequisites for the effective operation of a mobile cabin hospital. Mobile cabin hospitals in Wuhan implement a management model led by the district government, operated by medical institutions, and coordinated by other relevant units such as electric power and water affairs departments (35, 36). The district government and medical institutions appoint professional management personnel to form the mobile cabin hospital headquarter (37), which consists of an information management group, medical group, nursing group, nosocomial infections control group, logistics group, and other departments (Figure 2). Each department has clearly defined responsibilities and division of labor. This flat organizational structure simplifies the vertical management levels and is suitable for the temporarily established management team of the mobile cabin hospital (38).

### Management of Nosocomial Infections

Management of nosocomial infections is important to cut off the transmission route of COVID-19 and reduce cross-infection. A mobile cabin hospital should set up a nosocomial infections control team that conducts regular trainings to control hospital infections for all medical personnel and strengthens personal protection education for patients (39, 40). The training for medical personnel mainly includes zoning of the hospital, hand hygiene, wearing and unloading personal protective equipment (PPE), cleaning and disinfection knowledge, etc. Furthermore, the strategy of “three zones–two channels” should be strictly implemented, and the corresponding rules and regulations for controlling nosocomial infections should be formulated and followed (32, 41). Control measures for nosocomial infections mainly include personal protection, environmental health management, management of occupational exposure, management of discharged patients, and waste disposal.

① Personal protection: Before entering and leaving the hospital, the medical personnel should wash their hands and correctly wear and take off the PPE (42). All patients are required to wear masks, and their daily necessities are cleaned and disinfected (29). Moreover, all medical personnel and patients must undergo daily temperature monitoring (43).

② Environmental health management: Daily environmental disinfection of the air, ground, public facilities, and pollutants must be performed (43, 44). All the disinfection protocols conducted need to be recorded, including disinfection methods, disinfectant name, disinfectant concentration, disinfection frequency, and disinfection time (45).

③ Management of occupational exposure: Occupational exposure involves skin, mucosa, and respiratory exposure (42). A process for reporting and managing occupational exposure should be regulated, and the emergency management of risks, such as mask slipping or goggle loosening, should be standardized (40).

④ Management of discharged patients: All belongings should be terminally disinfected before the patient leaves the facility. Additionally, the clothing and daily necessities not taken away by the patients need to be treated as medical waste and handed over to the cleaners for centralized incineration (22).

⑤ Waste disposal: Mobile cabin hospitals produce a large amount of waste, such as medical waste, domestic waste, and patient excrement (feces, respiratory vomit, and other bodily secretions). All wastes generated in mobile cabin hospitals should be strictly managed (31). First, the collection, classification, packaging, sealing, marking, and treatment of waste should be regulated. Second, the temporary storage of waste, including storage time and disinfection methods, should be regulated. Third, the transshipment of waste, including transshipment time and route, vehicle selection, handover registration, and information sharing, should be clarified.

### Information Technology Support

The information technology support not only improves the work efficiency of the medical staff, but also reduces the risk of cross-infection in hospitals (46). In the case of a pandemic, several aspects can be improved by using state-of-the-art technology (47, 48).

① The establishment of electronic medical record systems, including the electronic medical records module in the desktop hospital information system (HIS) and mobile electronic medical record system, can improve the quality of medical records (49–51).

② The pharmacy information system is used for maintaining records of the drug supply in and out of the warehouse, drug data collection, drug planning, prescription deployment, and expiration period management (52–58). It can automatically generate a drug catalog that enables pharmacists to query drug consumption and inventory quickly.

③ Through the HIS remote consultation system, the medical teams inside and outside the mobile cabin hospital can record the patient's vital signs and changes in disease conditions and perform timely adjustment of the treatment plans based on the patient's condition (59).

④ The application of high-technological products can reduce the workload of the medical personnel. For example, smart wristbands and watches can be used to monitor the patient's blood oxygen saturation, heart rate, and blood pressure and upload the data to the cloud platform, thus facilitating remote monitoring of patients (60). Notably, the mobile cabin hospitals of Jianghan developed an online application in which patients can request life support, healthcare, and other services through their mobile phones, and the medical staffs can accept these requests online and provide services to meet the needs of the patients (16).

### Material Supply

The basis for the effective operation of mobile cabin hospitals is adequate material supplies, including medical equipment, medications, vaccines, PPE, and rapid diagnostic tests (61). The material supply of mobile cabin hospitals is coordinated and implemented by the government and medical institutions in China. To improve the effectiveness and timeliness of medical material support in China, a national information platform was built, which was mainly used to collect, analyze, monitor, and schedule the production, output, inventory, and transportation of various medical materials (62). The government mobilized manufacturing to ensure the supply chain of medical equipment and materials. Preferential financial and tax policies for supporting the prevention and control of the epidemic were issued, and the financial support for drug and vaccine research was increased (63). Furthermore, local government departments and many hospitals issued donation notices to the whole society, set up a lead group for donating materials, and gathered a large number of materials both from internal resources and abroad (64).

## Policy Implications

### Building Emergency Medical Rescue Team

To improve the emergency treatment function of medical institutions further, the construction of emergency medical rescue teams, especially personnel training to tackle public health emergencies, should be strengthened (65).

### Establishing Emergency Material Reserve Mechanism

To avoid the shortage of medical supplies such as drugs and PPE, during public health emergencies, the state should quickly deploy and establish an emergency reserve system for medical materials (62). Under the unified organization of the health administrative department, each medical institution formulates a material list according to the actual needs and then procures and stores the required medical emergency materials.

### Design and Construction of Large-Scale Public Venues

Converting large-scale public venues into mobile cabin hospitals is an important means of rapidly upgrading the healthcare system's capacity (10). Adaptability, convertibility, and expandability strategies should be included in the architectural design and construction planning of large-scale public buildings to allow for venues such as urban stadiums and exhibition centers to be converted into hospitals rapidly during major public health emergencies (25). For example, the interfaces of ventilation installation, sewage treatment systems and utilities should be reserved during architectural design and construction (4).

### Strengthening the Financial Support of the Public Health System

The COVID-19 crisis has highlighted the importance of a strong national public health system. A high-grade public health system needs sufficient funds; therefore, developing countries need to invest more in the healthcare sector to manage urgent health needs, such as establishing testing laboratories, setting up special wards, and procuring medical supplies (63, 66).

## Other Application Limitations to Be Concerned

The above experience has some limitations. First, this review aimed to summarize the emergency preparedness and management of the mobile cabin hospitals during the COVID-19 pandemic; hence, our findings may contribute only to the control of the transmission of respiratory pathogens rather than all pathogens. In the management of patients with other diseases that spread through contact and / or enteric transmission, such as cholera, bubonic plague, and Ebola, the design of the mobile cabin hospital should be modified accordingly. For instance, the distance mentioned here between two beds is based on whether it is an airborne or droplet-transmitted pathogen. Second, mobile cabin hospitals can easily be erected in high-income countries. However, in low- and middle-income countries, relative inadequacy of resources and infrastructure may not meet the requirements for setting up these cabins. Therefore, the reconstruction and management of mobile cabin hospitals in these countries should be simplified to avoid cross-infection. For instance, electronic medical records may be replaced with paper records in countries that lack electronic information management systems.

## Conclusion

Mobile cabin hospitals can be a key component of national public health responses to major epidemics, providing isolation and medical care for mild-to-moderate cases. Appropriate preparation and construction plans are necessary for converting large-scale public venues into mobile cabin hospitals, and a detailed management scheme is conducive for the normal operation of the hospital. This review may provide policymakers with useful information to upgrade the healthcare system's capacity by operating mobile cabin hospitals during the COVID-19 pandemic and provide a valuable reference for preparedness for any future such outbreaks.

## Author Contributions

CY, FS, and HL: conception and design and manuscript revision. FS, RL, YL, XL, and HW: literature research. FS and RL: first draft. All authors contributed to the article and approved the submitted version.

## Funding

This work was supported by the National Natural Science Foundation of China (grant numbers 81773552, 82173626) and the National Key Research and Development Program of China (grant numbers 2017YFC1200502, 2018YFC1315302).

## Conflict of Interest

The authors declare that the research was conducted in the absence of any commercial or financial relationships that could be construed as a potential conflict of interest.

## Publisher's Note

All claims expressed in this article are solely those of the authors and do not necessarily represent those of their affiliated organizations, or those of the publisher, the editors and the reviewers. Any product that may be evaluated in this article, or claim that may be made by its manufacturer, is not guaranteed or endorsed by the publisher.
