# Supplementary material for: Emergency Preparedness and Management of Mobile Cabin Hospitals in China During the COVID-19 Pandemic
Source: Front Public Health. 2022 Jan 3;9:763723. doi: 10.3389/fpubh.2021.763723 (PMC8761647; doi:10.3389/fpubh.2021.763723)
Supplement: Supplementary file 1 [file Table_1.DOCX]

Supplementary materials

Supplementary Table 1. Search strategies of databases.

| Databases | Search strategies | Search outcomes |
| --- | --- | --- |
| Pubmed  (https://pubmed.ncbi.nlm.nih.gov/advanced) | #1 All Fields: “Mobile Cabin Hospital” OR “Cabin Hospital” OR “Fangcang Shelter Hospital” OR “Shelter Hospital” OR “Mobile field hospital” OR “Ark Hospital” OR “Makeshift hospital”  #2 All Fields: COVID-19  #3 All Fields: China  #4 (#1 AND #2 AND #3)  Filters: Publication date to 2021/9/30 | 75 |
| IEEE  (https://ieeexplore.ieee.org/search/advanced) | #1 Full Text & Metadata: “Mobile Cabin Hospital” OR “Cabin Hospital” OR “Fangcang Shelter Hospital” OR “Shelter Hospital” OR “Mobile field hospital” OR “Ark Hospital” OR “Makeshift hospital”  #2 (#1 AND Full Text & Metadata: COVID-19)  #3 (#2 AND Full Text & Metadata: China)  Year Range: From 2020 To 2021  Filters Applied: Journals and Books | 115 |
| CNKI  (https://www.cnki.net/) | #1主题: 方舱医院 (Chinese expression of mobile cabin hospital)  #2主题: 新型冠状病毒 + 新冠肺炎 (Chinese expression of COVID-19)  #3 (#1 AND #2)  Year Range: From 2020-02-04 To 2021/9/30 | 317 |

Supplementary Table 2. Information of the retrieved literature and the reasons for exclusion.

| ID | Database | Author | Title | Journal / URL | Reason for literature exclusion |
| --- | --- | --- | --- | --- | --- |
| 1 | Pubmed | Chen S, Sun H, Heng M, Tong X, Geldsetzer P, Wang Z, Wu P, Yang J, Hu Y, Wang C, Bärnighausen T. | Factors Predicting Progression to Severe COVID-19: A Competing Risk Survival Analysis of 1753 Patients in Community Isolation in Wuhan, China. | Engineering (Beijing) | 6 |
| 2 | Pubmed | Lu J, Zhao M, Wu Q, Ma C, Du X, Lu X, Jia Q, Li C. | Mental health needs of the COVID-19 patients and staff in the Fangcang shelter hospital: a qualitative research in Wuhan, China. | Glob Ment Health (Camb) | 4 |
| 3 | Pubmed | Li M, Zu J, Li Z, Shen M, Li Y, Ji F. | How to Reduce the Transmission Risk of COVID-19 More Effectively in New York City: An Age-Structured Model Study. | Front Med (Lausanne). | 6 |
| 4 | Pubmed | Zhang XB, Zhang JL, Li MX, Yuan YP, Sun J. | Baduanjin exercise can alleviate anxiety and depression of patients with COVID-19 in Square cabin hospital: A cross-sectional survey. | Medicine (Baltimore). | 4 |
| 5 | Pubmed | Wang Y, Zhang M, Yin Q, Wang Y, Yang P, Hu C, Xu G, Wang D, Li X, He J, Hu Q, Luo X, Ren H. | Psychological Responses of the Patients in Cabin Hospital to the COVID-19 Outbreak: A Comparative Epidemiologic Analysis. | Front Psychol. | 4 |
| 6 | Pubmed | Mi X, Li H, Tan R, Feng B, Tu Y. | The TDs/aptamer cTnI biosensors based on HCR and Au/Ti 3 C 2-MXene amplification for screening serious patient in COVID-19 pandemic. | Biosens Bioelectron. | 6 |
| 7 | Pubmed | Fan S, Zhen Q, Chen C, Wang W, Wu Q, Ma H, Zhang C, Zhang L, Lu B, Ge H, Yong L, Li B, Yu Y, Chen W, Mao Y, Qu G, Su L, Wang A, Ding Z, Li H, Zhang J, Wang Y, Gao Y, Xu X, Zhu Z, Chen J, Zhang L, Liang H, Wu S, Huang M, Xia Q, Li P, Sun Y, Liang C, Wei W, Liu Q, Sun L. | Clinical efficacy of low-dose emetine for patients with COVID-19: a real-world study. | J BioX Res. | 2 |
| 8 | Pubmed | Jiang H, Song P, Wang S, Yin S, Yin J, Zhu C, Cai C, Xu W, Li W. | Quantitative assessment of the effectiveness of joint measures led by Fangcang shelter hospitals in response to COVID-19 epidemic in Wuhan, China. | BMC Infect Dis. | 0 |
| 9 | Pubmed | Lv Y, Zhao X, Wang Y, Zhu J, Ma C, Feng X, Ma Y, Zheng Y, Yang L, Han G, Xie H. | Abnormal Liver Function Tests Were Associated With Adverse Clinical Outcomes: An Observational Cohort Study of 2,912 Patients With COVID-19. | Front Med (Lausanne). | 2 |
| 10 | Pubmed | Zhang Y, Shi L, Chen H, Wang X, Sun G. | Hubei's Core Response Policies in the Early Stage of COVID-19. | Biomed Res Int. | 6 |
| 11 | Pubmed | Mu L, Zhang C, Pei Y, Wang J. | The worldwide coronavirus disease 2019 outbreak: Advice and recommendation on radiology management and infection control from makeshift hospitals in Wuhan. | Medicine (Baltimore). | 5 |
| 12 | Pubmed | Zhang XB, Xiao W, Lei J, Li MX, Wang X, Hong YJ, Xu P, Sun J. | Prevalence and influencing factors of anxiety and depression symptoms among the first-line medical staff in Wuhan mobile cabin hospital during the COVID-19 epidemic: A cross-sectional survey. | Medicine (Baltimore). | 4 |
| 13 | Pubmed | Tian JD, Xie M, Wen ZC, Xu JM, Wen C. | [Epidemiological and clinical features of children with mild coronavirus disease 2019]. | Zhongguo Dang Dai Er Ke Za Zhi. | 6 |
| 14 | Pubmed | Dong L, Chen L, Ding S. | Illness uncertainty among patients with COVID-19 in the Mobile Cabin Hospital. | Nurs Open. | 2 |
| 15 | Pubmed | Zhang X, Huang DS, Guan P. | Nursing Scheduling Mode and Experience from the Medical Teams in Aiding Hubei Province During the COVID-19 Outbreak: A Systematic Scoping Review of 17 Studies. | Risk Manag Healthc Policy. | 3 |
| 16 | Pubmed | Chen X, Chen H, Chen YG, Yao JG. | Cabin hospital for COVID-19 patients: Attention should be paid to hospital infection prevention, humanistic care, and privacy protection. | Kaohsiung J Med Sci. | 5 |
| 17 | Pubmed | He J, Yang L, Pang J, Dai L, Zhu J, Deng Y, He Y, Li H. | Efficacy of simplified-cognitive behavioral therapy for insomnia(S-CBTI) among female COVID-19 patients with insomnia symptom in Wuhan mobile cabin hospital. | Sleep Breath. | 4 |
| 18 | Pubmed | Huang Z, Zhao S, Leng Q, Hu S, Li Z, Song B. | Container CT scanner: a solution for modular emergency radiology department during the COVID-19 pandemic. | Diagn Interv Radiol. | 2 |
| 19 | Pubmed | Li Z, Ge J, Feng J, Jiang R, Zhou Q, Xu X, Pan Y, Liu S, Gui B, Wang Z, Zhu B, Hu Y, Yang J, Wang R, Su D, Hashimoto K, Yang M, Yang C, Liu C. | Less Social Support for Patients With COVID-19: Comparison With the Experience of Nurses. | Front Psychiatry. | 4 |
| 20 | Pubmed | He Q, Xiao H, Li HM, Zhang BB, Li CW, Yuan FJ, Yu SS, Zhang F, Kong P. | Practice in Information Technology Support for Fangcang Shelter Hospital during COVID-19 Epidemic in Wuhan, China. | J Med Syst. | 0 |
| 21 | Pubmed | Zhang Y, Shi L, Cao Y, Chen H, Wang X, Sun G. | Wuhan mobile cabin hospital: A critical health policy at a critical time in China. | Medicine (Baltimore). | 0 |
| 22 | Pubmed | Yan B, Song L, Guo J, Wang Y, Peng L, Li D. | Association Between Clinical Characteristics and Short-Term Outcomes in Adult Male COVID-19 Patients With Mild Clinical Symptoms: A Single-Center Observational Study. | Front Med (Lausanne). | 2 |
| 23 | Pubmed | Zheng Y, Hu R, Zhang L, Li S. | [Follow-up results of 117 discharged patients with coronavirus disease 2019 in Wuhan Mobile Cabin Hospital]. | Zhonghua Wei Zhong Bing Ji Jiu Yi Xue. | 2 |
| 24 | Pubmed | Lu C, Wang HY, Chen X, Wu ZL, Meng S, He W, Xu KS, Fu XQ, Hu Y, Yang C, Sun H. | Clinical characteristics of 1327 patients with coronavirus disease 2019 (COVID-19) in the largest Fangcang shelter hospital in Wuhan. | Chin Med J (Engl). | 2 |
| 25 | Pubmed | Lv Z, Lv S. | Clinical characteristics and analysis of risk factors for disease progression of COVID-19: A retrospective Cohort Study. | Int J Biol Sci. | 2 |
| 26 | Pubmed | Sun Y, Guan X, Jia L, Xing N, Cheng L, Liu B, Zhang S, He K. | Independent and combined effects of hypertension and diabetes on clinical outcomes in patients with COVID-19: A retrospective cohort study of Huoshen Mountain Hospital and Guanggu Fangcang Shelter Hospital. | J Clin Hypertens (Greenwich). | 2 |
| 27 | Pubmed | Meng Q, Liu W, Gao P, Zhang J, Sun A, Ding J, Liu H, Lei Z. | Novel Deep Learning Technique Used in Management and Discharge of Hospitalized Patients with COVID-19 in China. | Ther Clin Risk Manag. | 6 |
| 28 | Pubmed | Horchinbilig U, Gao Y, Chang H, Xi P, Wu J, Wang J, Liu W. | Investigation of 100 SARS-CoV-2 infected families in Wuhan: Transmission patterns and follow-up. | J Glob Health. | 6 |
| 29 | Pubmed | Gu Y, Zhu Y, Xu G. | Factors associated with mental health outcomes among health care workers in the Fangcang shelter hospital in China. | Int J Soc Psychiatry. | 4 |
| 30 | Pubmed | Sun H, Wang S, Wang W, Han G, Liu Z, Wu Q, Pang X. | Correlation between emotional intelligence and negative emotions of front-line nurses during the COVID-19 epidemic: A cross-sectional study. | J Clin Nurs. | 4 |
| 31 | Pubmed | Gu Y, Zhu Y, Xu F, Xi J, Xu G. | Factors associated with mental health outcomes among patients with COVID-19 treated in the Fangcang shelter hospital in China. | Asia Pac Psychiatry. | 4 |
| 32 | Pubmed | Cheng F, Li Q, Han Y, Shi C, Wu S, Xu Q, Zeng F, Zhang Y. | Analysis of Influencing Factors and Pharmaceutical Care of Patients with COVID-19 in Fangcang Shelter Hospital. | Infect Drug Resist. | 2 |
| 33 | Pubmed | Gao W, Chen S, Wang K, Chen R, Guo Q, Lu J, Wu X, He Y, Yan Q, Wang S, Wang F, Jin L, Hua J, Li Q. | Clinical features and efficacy of antiviral drug, Arbidol in 220 nonemergency COVID-19 patients from East-West-Lake Shelter Hospital in Wuhan: a retrospective case series. | Virol J. | 2 |
| 34 | Pubmed | Wu S, Xue L, Legido-Quigley H, Khan M, Wu H, Peng X, Li X, Li P. | Understanding factors influencing the length of hospital stay among non-severe COVID-19 patients: A retrospective cohort study in a Fangcang shelter hospital. | PLoS One. | 2 |
| 35 | Pubmed | Cai Q, Du SY, Gao S, Huang GL, Zhang Z, Li S, Wang X, Li PL, Lv P, Hou G, Zhang LN. | A model based on CT radiomic features for predicting RT-PCR becoming negative in coronavirus disease 2019 (COVID-19) patients. | BMC Med Imaging. | 2 |
| 36 | Pubmed | Liao Y, Feng Y, Wang B, Wang H, Huang J, Wu Y, Wu Z, Chen X, Yang C, Fu X, Sun H. | Clinical characteristics and prognostic factors of COVID-19 patients progression to severe: a retrospective, observational study. | Aging (Albany NY). | 2 |
| 37 | Pubmed | Liu P, Zhang H, Long X, Wang W, Zhan D, Meng X, Li D, Wang L, Chen R. | Management of COVID-19 patients in Fangcang shelter hospital: clinical practice and effectiveness analysis. | Clin Respir J. | 0 |
| 38 | Pubmed | Wang M, Xia C, Huang L, Xu S, Qin C, Liu J, Cao Y, Yu P, Zhu T, Zhu H, Wu C, Zhang R, Chen X, Wang J, Du G, Zhang C, Wang S, Chen K, Liu Z, Xia L, Wang W. | Deep learning-based triage and analysis of lesion burden for COVID-19: a retrospective study with external validation. | Lancet Digit Health. | 2 |
| 39 | Pubmed | Yan N, Xu Z, Mei B, Gao Y, Lv D, Zhang J. | Neurological Implications of Non- critically Ill Patients With Coronavirus Disease 2019 in a Fangcang Shelter Hospital in Wuhan, China. | Front Neurol. | 2 |
| 40 | Pubmed | Liu YS, Peng DL, Yang J, Chen DY, Jia HB, Yu SY, Chen HH, Chen K, Liu LR. | Emergency Biosafety Management Practice in Laboratory of Shelter Hospital. | Curr Med Sci. | 5 |
| 41 | Pubmed | Cai Z, Cui Q, Liu Z, Li J, Gong X, Liu J, Wan Z, Yuan X, Li X, Chen C, Wang G. | Nurses endured high risks of psychological problems under the epidemic of COVID-19 in a longitudinal study in Wuhan China. | J Psychiatr Res. | 4 |
| 42 | Pubmed | Zhang H, Qin S, Zhang L, Feng Z, Fan C. | A psychological investigation of coronavirus disease 2019 (COVID-19) patients in mobile cabin hospitals in Wuhan. | Ann Transl Med. | 4 |
| 43 | Pubmed | Guo T, Liu X, Xu C, Wang J, Yang L, Shi H, Dai M. | Fangcang Shelter Hospital in Wuhan: A radiographic report on a cohort of 98 COVID-19 patients. | Int J Med Sci. | 2 |
| 44 | Pubmed | Dai LL, Wang X, Jiang TC, Li PF, Wang Y, Wu SJ, Jia LQ, Liu M, An L, Cheng Z. | Anxiety and depressive symptoms among COVID-19 patients in Jianghan Fangcang Shelter Hospital in Wuhan, China. | PLoS One. | 4 |
| 45 | Pubmed | Shen B, Chen L, Zhang L, Zhang M, Li J, Wu J, Chen K, Xiong Y, Song W, Zhou B. | Wuchang Fangcang Shelter Hospital: Practices, Experiences, and Lessons Learned in Controlling COVID-19. | SN Compr Clin Med. | 5 |
| 46 | Pubmed | Liu X, Chang YC. | An emergency responding mechanism for cruise epidemic prevention-taking COVID-19 as an example. | Mar Policy. | 6 |
| 47 | Pubmed | Shu L, Wang X, Li M, Chen X, Ji N, Shi L, Wu M, Deng K, Wei J, Wang X, Cao Y, Yan J, Feng G. | Clinical characteristics of moderate COVID-19 patients aggravation in Wuhan Stadium Cabin Hospital: A 571 cases of retrospective cohort study. | J Med Virol. | 2 |
| 48 | Pubmed | Li Y, Shi J, Xia J, Duan J, Chen L, Yu X, Lan W, Ma Q, Wu X, Yuan Y, Gong L, Yang X, Gao H, Wu C. | Asymptomatic and Symptomatic Patients With Non-severe Coronavirus Disease (COVID-19) Have Similar Clinical Features and Virological Courses: A Retrospective Single Center Study. | Front Microbiol. | 2 |
| 49 | Pubmed | Naganathan S, Meehan-Coussee K, Pasichow S, Rybasack-Smith H, Binder W, Beaudoin F, Musits AN, Sutton E, Petrone G, Levine AC, Suner S. | From Concerts to COVID: Transforming the RI Convention Center into an Alternate Hospital Site in under a Month. | R I Med J (2013). | 6 |
| 50 | Pubmed | Zhang M, Wang L, Yu S, Sun G, Lei H, Wu W. | Status of occupational protection in the COVID-19 Fangcang Shelter Hospital in Wuhan, China. | Emerg Microbes Infect. | 0 |
| 51 | Pubmed | Yan N, Wang W, Gao Y, Zhou J, Ye J, Xu Z, Cao J, Zhang J. | Medium Term Follow-Up of 337 Patients With Coronavirus Disease 2019 (COVID-19) in a Fangcang Shelter Hospital in Wuhan, China. | Front Med (Lausanne). | 2 |
| 52 | Pubmed | Ding T, Zhang J, Wang T, Cui P, Chen Z, Jiang J, Zhou S, Dai J, Wang B, Yuan S, Ma W, Ma L, Rong Y, Chang J, Miao X, Ma X, Wang S. | Potential Influence of Menstrual Status and Sex Hormones on Female Severe Acute Respiratory Syndrome Coronavirus 2 Infection: A Cross-sectional Multicenter Study in Wuhan, China. | Clin Infect Dis. | 2 |
| 53 | Pubmed | Wang B, Wang Z, Zhao J, Zeng X, Wu M, Wang S, Wang T. | Epidemiological and clinical course of 483 patients with COVID-19 in Wuhan, China: a single-center, retrospective study from the mobile cabin hospital. | Eur J Clin Microbiol Infect Dis. | 2 |
| 54 | Pubmed | Zhao ZH, Zhou Y, Li WH, Huang QS, Tang ZH, Li H. | Analysis of Traditional Chinese Medicine Diagnosis and Treatment Strategies for COVID-19 Based on "The Diagnosis and Treatment Program for Coronavirus Disease-2019" from Chinese Authority. | Am J Chin Med. | 2 |
| 55 | Pubmed | Li Q, Wang H, Li X, Zheng Y, Wei Y, Zhang P, Ding Q, Lin J, Tang S, Zhao Y, Zhao L, Tong X. | The role played by traditional Chinese medicine in preventing and treating COVID-19 in China | Front Med. | 2 |
| 56 | Pubmed | Zhang D, Ling H, Huang X, Li J, Li W, Yi C, Zhang T, Jiang Y, He Y, Deng S, Zhang X, Wang X, Liu Y, Li G, Qu J. | Potential spreading risks and disinfection challenges of medical wastewater by the presence of Severe Acute Respiratory Syndrome Coronavirus 2 (SARS-CoV-2) viral RNA in septic tanks of Fangcang Hospital. | Sci Total Environ. | 0 |
| 57 | Pubmed | Zhang J, Wang M, Zhao M, Guo S, Xu Y, Ye J, Ding W, Wang Z, Ye D, Pan W, Liu M, Li D, Luo Z, Liu J, Wan J. | The Clinical Characteristics and Prognosis Factors of Mild-Moderate Patients With COVID-19 in a Mobile Cabin Hospital: A Retrospective, Single-Center Study. | Front Public Health. | 2 |
| 58 | Pubmed | Zheng T, Yang C, Wang HY, Chen X, Yu L, Wu ZL, Sun H. | Clinical characteristics and outcomes of COVID-19 patients with gastrointestinal symptoms admitted to Jianghan Fangcang Shelter Hospital in Wuhan, China. | J Med Virol. | 2 |
| 59 | Pubmed | Wu J, Shen B, Li D, Song W, Li J, Zhang M, Liu G, Zhou B. | Pharmacy services at a temporary COVID-19 hospital in Wuhan, China. | Am J Health Syst Pharm. | 5 |
| 60 | Pubmed | Zhou B, Wu Q, Zhao X, Zhang W, Wu W, Guo Z. | Construction of 5G all-wireless network and information system for cabin hospitals. | J Am Med Inform Assoc. | 0 |
| 61 | Pubmed | Wang W, Xin C, Xiong Z, Yan X, Cai Y, Zhou K, Xie C, Zhang T, Wu X, Liu K, Li Z, Chen J. | Clinical Characteristics and Outcomes of 421 Patients With Coronavirus Disease 2019 Treated in a Mobile Cabin Hospital. | Chest. | 2 |
| 62 | Pubmed | Chen L, Deng C, Chen X, Zhang X, Chen B, Yu H, Qin Y, Xiao K, Zhang H, Sun X. | Ocular manifestations and clinical characteristics of 535 cases of COVID-19 in Wuhan, China: a cross-sectional study. | Acta Ophthalmol. | 2 |
| 63 | Pubmed | Zhang T, Ran L, Li K, Liu J, Chen L, Lü J, Liang G, Zuo S. | [Supporting the battle against COVID-19 in E'zhou, Hubei Province: the experience of Guizhou medical team]. | Nan Fang Yi Ke Da Xue Xue Bao. | 6 |
| 64 | Pubmed | Shang L, Xu J, Cao B. | Fangcang shelter hospitals in COVID-19 pandemic: the practice and its significance. | Clin Microbiol Infect. | 0 |
| 65 | Pubmed | Sun C, Wu Q, Zhang C. | Managing patients with COVID-19 infections: a first- hand experience from the Wuhan Mobile Cabin Hospital. | Br J Gen Pract. | 7 |
| 66 | Pubmed | Yuan Y, Qiu T, Wang T, Zhou J, Ma Y, Liu X, Deng H. | The application of Temporary Ark Hospitals in controlling COVID-19 spread: The experiences of one Temporary Ark Hospital, Wuhan, China. | J Med Virol. | 0 |
| 67 | Pubmed | Wu S, Qiao R, Wang C. | PCCM at the Battlefront Against COVID-19 in Wuhan, China. | Chest. | 6 |
| 68 | Pubmed | Zhou F, Gao X, Li M, Zhang Y. | Shelter Hospital: Glimmers of Hope in Treating Coronavirus 2019. | Disaster Med Public Health Prep. | 0 |
| 69 | Pubmed | COVID-19 Emergency Response Key Places Protection and Disinfection Technology Team, Chinese Center for Disease Control and Prevention. | [Health protection guideline of mobile cabin hospitals during COVID-19 outbreak]. | Zhonghua Yu Fang Yi Xue Za Zhi. | 0 |
| 70 | Pubmed | Ma SY, Luo YM, Hu TY, You ZC, Sun JG, Yu SY, Yuan ZQ, Peng YZ, Luo GX, Xu Z. | [Clinical application effect of modified nasopharyngeal swab sampling for 2019 novel coronavirus nucleic acid detection]. | Zhonghua Shao Shang Za Zhi. | 2 |
| 71 | Pubmed | Shu L, Ji N, Chen X, Feng G. | Ark of Life and Hope: the role of the Cabin Hospital in facing COVID-19. | J Hosp Infect. | 7 |
| 72 | Pubmed | Meng L, Qiu F, Sun S. | Providing pharmacy services at cabin hospitals at the coronavirus epicenter in China. | Int J Clin Pharm. | 0 |
| 73 | Pubmed | Chen Z, He S, Li F, Yin J, Chen X. | Mobile field hospitals, an effective way of dealing with COVID-19 in China: sharing our experience. | Biosci Trends. | 0 |
| 74 | Pubmed | Yang Y, Wang H, Chen K, Zhou J, Deng S, Wang Y. | Shelter hospital mode: How do we prevent COVID-19 hospital-acquired infection? | Infect Control Hosp Epidemiol. | 0 |
| 75 | Pubmed | Chen S, Huang B, Luo DJ, Li X, Yang F, Zhao Y, Nie X, Huang BX. | [Pregnancy with new coronavirus infection: clinical characteristics and placental pathological analysis of three cases]. | Zhonghua Bing Li Xue Za Zhi. | 2 |
| 76 | IEEE | S. Wu; D. Wu; R. Ye; K. Li; Y. Lu; J. Xu; L. Xiong; Y. Zhao; A. Cui; Y. Li; C. Peng; F. Lv | Pilot Study of Robot-Assisted Teleultrasound Based on 5G Network: A New Feasible Strategy for Early Imaging Assessment During COVID-19 Pandemic | IEEE Transactions on Ultrasonics, Ferroelectrics, and Frequency Control | 6 |
| 77 | IEEE | X. Ding; D. Clifton; N. Ji; N. H. Lovell; P. Bonato; W. Chen; X. Yu; Z. Xue; T. Xiang; X. Long; K. Xu; X. Jiang; Q. Wang; B. Yin; G. Feng; Y. -T. Zhang | Wearable Sensing and Telehealth Technology with Potential Applications in the Coronavirus Pandemic | IEEE Reviews in Biomedical Engineering | 0 |
| 78 | IEEE | M. Marinelli | Emergency Healthcare Facilities: Managing Design in a Post Covid-19 World | IEEE Engineering Management Review | 0 |
| 79 | IEEE | M. Berquedich; A. Berquedich; O. Kamach; M. Masmoudi; A. Chebbak; L. Deshayes | Developing a Mobile COVID-19 Prototype Management Application Integrated With an Electronic Health Record for Effective Management in Hospitals | IEEE Engineering Management Review | 6 |
| 80 | IEEE | J. Chen; C. He; J. Yin; J. Li; X. Duan; Y. Cao; L. Sun; M. Hu; W. Li; Q. Li | Quantitative Analysis and Automated Lung Ultrasound Scoring for Evaluating COVID-19 Pneumonia With Neural Networks | IEEE Transactions on Ultrasonics, Ferroelectrics, and Frequency Control | 6 |
| 81 | IEEE | B. Gharizadeh; J. Yue; M. Yu; Y. Liu; M. Zhou; D. Lu; J. Zhang | Navigating the Pandemic Response Life Cycle: Molecular Diagnostics and Immunoassays in the Context of COVID-19 Management | IEEE Reviews in Biomedical Engineering | 6 |
| 82 | IEEE | R. Yin; G. He; W. Jiang; Y. Peng; Z. Zhang; M. Li; C. Gong | Night-Time Light Imagery Reveals China's City Activity During the COVID-19 Pandemic Period in Early 2020 | IEEE Journal of Selected Topics in Applied Earth Observations and Remote Sensing | 6 |
| 83 | IEEE | M. T. Rahman; R. T. Khan; M. R. A. Khandaker; M. Sellathurai; M. S. A. Salan | An Automated Contact Tracing Approach for Controlling Covid-19 Spread Based on Geolocation Data From Mobile Cellular Networks | IEEE Access | 6 |
| 84 | IEEE | K. Qian; M. Schmitt; H. Zheng; T. Koike; J. Han; J. Liu; W. Ji; J. Duan; M. Song; Z. Yang; Z. Ren; S. Liu; Z. Zhang; Y. Yamamoto; B. W. Schuller | Computer Audition for Fighting the SARS-CoV-2 Corona Crisis—Introducing the Multitask Speech Corpus for COVID-19 | IEEE Internet of Things Journal | 6 |
| 85 | IEEE | N. Paluru; A. Dayal; H. B. Jenssen; T. Sakinis; L. R. Cenkeramaddi; J. Prakash; P. K. Yalavarthy | Anam-Net: Anamorphic Depth Embedding-Based Lightweight CNN for Segmentation of Anomalies in COVID-19 Chest CT Images | IEEE Transactions on Neural Networks and Learning Systems | 6 |
| 86 | IEEE | X. Song; H. Li; W. Gao; Y. Chen; T. Wang; G. Ma; B. Lei | Augmented Multicenter Graph Convolutional Network for COVID-19 Diagnosis | IEEE Transactions on Industrial Informatics | 6 |
| 87 | IEEE | F. Shi; J. Wang; J. Shi; Z. Wu; Q. Wang; Z. Tang; K. He; Y. Shi; D. Shen | Review of Artificial Intelligence Techniques in Imaging Data Acquisition, Segmentation, and Diagnosis for COVID-19 | IEEE Reviews in Biomedical Engineering | 6 |
| 88 | IEEE | X. Kong; K. Wang; S. Wang; X. Wang; X. Jiang; Y. Guo; G. Shen; X. Chen; Q. Ni | Real-Time Mask Identification for COVID-19: An Edge-Computing-Based Deep Learning Framework | IEEE Internet of Things Journal | 6 |
| 89 | IEEE | F. Firouzi; B. Farahani; M. Daneshmand; K. Grise; J. Song; R. Saracco; L. L. Wang; K. Lo; P. Angelov; E. Soares; P. -S. Loh; Z. Talebpour; R. Moradi; M. Goodarzi; H. Ashraf; M. Talebpour; A. Talebpour; L. Romeo; R. Das; H. Heidari; D. Pasquale; J. Moody; C. Woods; E. S. Huang; P. Barnaghi; M. Sarrafzadeh; R. Li; K. L. Beck; O. Isayev; N. Sung; A. Luo | Harnessing the Power of Smart and Connected Health to Tackle COVID-19: IoT, AI, Robotics, and Blockchain for a Better World | IEEE Internet of Things Journal | 6 |
| 90 | IEEE | S. H. Alsamhi; B. Lee | Blockchain-Empowered Multi-Robot Collaboration to Fight COVID-19 and Future Pandemics | IEEE Access | 6 |
| 91 | IEEE | S. Yu; Q. Qing; C. Zhang; A. Shehzad; G. Oatley; F. Xia | Data-Driven Decision-Making in COVID-19 Response: A Survey | IEEE Transactions on Computational Social Systems | 6 |
| 92 | IEEE | K. Song; S. Jiao; Q. Zhu; H. Wu | A Proactive and Practical COVID-19 Testing Strategy | IEEE Engineering Management Review | 6 |
| 93 | IEEE | S. Sakib; T. Tazrin; M. M. Fouda; Z. M. Fadlullah; M. Guizani | DL-CRC: Deep Learning-Based Chest Radiograph Classification for COVID-19 Detection: A Novel Approach | IEEE Access | 6 |
| 94 | IEEE | V. Chamola; V. Hassija; V. Gupta; M. Guizani | A Comprehensive Review of the COVID-19 Pandemic and the Role of IoT, Drones, AI, Blockchain, and 5G in Managing its Impact | IEEE Access | 6 |
| 95 | IEEE | S. Kumar; R. Viral | Effect, Challenges, and Forecasting of COVID-19 Situation in India Using an ARMA Model | IEEE Transactions on Computational Social Systems | 6 |
| 96 | IEEE | L. Garg; E. Chukwu; N. Nasser; C. Chakraborty; G. Garg | Anonymity Preserving IoT-Based COVID-19 and Other Infectious Disease Contact Tracing Model | IEEE Access | 6 |
| 97 | IEEE | Y. Tai; B. Gao; Q. Li; Z. Yu; C. Zhu; V. Chang | Trustworthy and Intelligent COVID-19 Diagnostic IoMT Through XR and Deep-Learning-Based Clinic Data Access | IEEE Internet of Things Journal | 6 |
| 98 | IEEE | Y. Zhang; Y. Li; B. Yang; X. Zheng; M. Chen | Risk Assessment of COVID-19 Based on Multisource Data From a Geographical Viewpoint | IEEE Access | 6 |
| 99 | IEEE | Y. Dong; Y. -D. Yao | IoT Platform for COVID-19 Prevention and Control: A Survey | IEEE Access | 6 |
| 100 | IEEE | B. Wang; Y. Zhao; C. L. P. Chen | Hybrid Transfer Learning and Broad Learning System for Wearing Mask Detection in the COVID-19 Era | IEEE Transactions on Instrumentation and Measurement | 6 |
| 101 | IEEE | M. Mahmeen; M. R. Melconian; S. Haider; M. Friebe; M. Pech | Next Generation 5G Mobile Health Network for User Interfacing in Radiology Workflows | IEEE Access | 6 |
| 102 | IEEE | H. Qiu; Y. Chen; S. Ding; W. Yi; R. Lv; C. Wang | An Improved Agent-Based Model Using Discrete Event Simulation for Nonpharmaceutical Interventions | IEEE Access | 6 |
| 103 | IEEE | K. D. Prashant | 5 Mobile Robots in COVID-19 | Use of AI, Robotics, and Modern Tools to Fight Covid-19 | 6 |
| 104 | IEEE | C. S. Meera; B. Aslesha; S. S. Pinisetti | 4 Technological Opportunities to Fight COVID-19 for Indian Scenario | Use of AI, Robotics, and Modern Tools to Fight Covid-19 | 6 |
| 105 | IEEE | Q. -V. Pham; D. C. Nguyen; T. Huynh-The; W. -J. Hwang; P. N. Pathirana | Artificial Intelligence (AI) and Big Data for Coronavirus (COVID-19) Pandemic: A Survey on the State-of-the-Arts | IEEE Access | 6 |
| 106 | IEEE | T. Zhang; M. Liu; T. Yuan; N. Al-Nabhan | Emotion-Aware and Intelligent Internet of Medical Things Toward Emotion Recognition During COVID-19 Pandemic | IEEE Internet of Things Journal | 6 |
| 107 | IEEE | M. Poongodi; M. Malviya; M. Hamdi; H. T. Rauf; S. Kadry; O. Thinnukool | The Recent Technologies to Curb the Second-Wave of COVID-19 Pandemic | IEEE Access | 6 |
| 108 | IEEE | A. Ulhaq; J. Born; A. Khan; D. P. S. Gomes; S. Chakraborty; M. Paul | COVID-19 Control by Computer Vision Approaches: A Survey | IEEE Access | 6 |
| 109 | IEEE | M. M. Islam; F. Karray; R. Alhajj; J. Zeng | A Review on Deep Learning Techniques for the Diagnosis of Novel Coronavirus (COVID-19) | IEEE Access | 6 |
| 110 | IEEE | E. Jordan; D. E. Shin; S. Leekha; S. Azarm | Optimization in the Context of COVID-19 Prediction and Control: A Literature Review | IEEE Access | 6 |
| 111 | IEEE | Y. -L. Zhao; H. -P. Huang; T. -L. Chen; P. -C. Chiang; Y. -H. Chen; J. -H. Yeh; C. -H. Huang; J. -F. Lin; W. -T. Weng | A Smart Sterilization Robot System With Chlorine Dioxide for Spray Disinfection | IEEE Sensors Journal | 6 |
| 112 | IEEE | M. J. M. Chowdhury; M. S. Ferdous; K. Biswas; N. Chowdhury; V. Muthukkumarasamy | COVID-19 Contact Tracing: Challenges and Future Directions | IEEE Access | 6 |
| 113 | IEEE | Y. Shen; D. Guo; F. Long; L. A. Mateos; H. Ding; Z. Xiu; R. B. Hellman; A. King; S. Chen; C. Zhang; H. Tan | Robots Under COVID-19 Pandemic: A Comprehensive Survey | IEEE Access | 6 |
| 114 | IEEE | M. Ndiaye; S. S. Oyewobi; A. M. Abu-Mahfouz; G. P. Hancke; A. M. Kurien; K. Djouani | IoT in the Wake of COVID-19: A Survey on Contributions, Challenges and Evolution | IEEE Access | 6 |
| 115 | IEEE | Q. Li; Z. Tang; N. Coleman; A. Mostafavi | Detecting Early-Warning Signals in Time Series of Visits to Points of Interest to Examine Population Response to COVID-19 Pandemic | IEEE Access | 6 |
| 116 | IEEE | G. Subramanian; A. S. Thampy; N. V. Ugwuoke; B. Ramnani | Crypto Pharmacy – Digital Medicine: A Mobile Application Integrated With Hybrid Blockchain to Tackle the Issues in Pharma Supply Chain | IEEE Open Journal of the Computer Society | 6 |
| 117 | IEEE | M. N. Islam; T. T. Inan; S. Rafi; S. S. Akter; I. H. Sarker; A. K. M. N. Islam | A Systematic Review on the Use of AI and ML for Fighting the COVID-19 Pandemic | IEEE Transactions on Artificial Intelligence | 6 |
| 118 | IEEE | X. Xu; S. A. Wala; A. Vishwa; J. Shen; D. K; S. Devi; A. Chandak; S. Dixit; E. Granata; S. Pithadia; V. Nimran; S. Oswal | A Programmable Platform for Accelerating the Development of Smart Ultrasound Transducer Probe | IEEE Transactions on Ultrasonics, Ferroelectrics, and Frequency Control | 6 |
| 119 | IEEE | M. N. Islam; A. K. M. N. Islam | A Systematic Review of the Digital Interventions for Fighting COVID-19: The Bangladesh Perspective | IEEE Access | 6 |
| 120 | IEEE | A. Castiglione; M. Umer; S. Sadiq; M. S. Obaidat; P. Vijayakumar | The Role of Internet of Things to Control the Outbreak of COVID-19 Pandemic | IEEE Internet of Things Journal | 6 |
| 121 | IEEE | A. Agarwal; D. Uniyal; D. Toshniwal; D. Deb | Dense Vector Embedding Based Approach to Identify Prominent Disseminators From Twitter Data Amid COVID-19 Outbreak | IEEE Transactions on Emerging Topics in Computational Intelligence | 6 |
| 122 | IEEE | M. A. Ferrag; L. Shu; K. -K. R. Choo | Fighting COVID-19 and Future Pandemics With the Internet of Things: Security and Privacy Perspectives | IEEE/CAA Journal of Automatica Sinica | 6 |
| 123 | IEEE | S. Temiz; D. G. Broo | Open Innovation Initiatives to Tackle COVID-19 Crises: Imposter Open Innovation and Openness in Data | IEEE Engineering Management Review | 6 |
| 124 | IEEE | N. Pathak; P. K. Deb; A. Mukherjee; S. Misra | IoT-to-the-Rescue: A Survey of IoT Solutions for COVID-19-Like Pandemics | IEEE Internet of Things Journal | 6 |
| 125 | IEEE | N. Y. Ahn; J. E. Park; D. H. Lee; P. C. Hong | Balancing Personal Privacy and Public Safety During COVID-19: The Case of South Korea | IEEE Access | 6 |
| 126 | IEEE | D. C. Nguyen; M. Ding; P. N. Pathirana; A. Seneviratne | Blockchain and AI-Based Solutions to Combat Coronavirus (COVID-19)-Like Epidemics: A Survey | IEEE Access | 6 |
| 127 | IEEE | S. Latif; M. Usman; S. Manzoor; W. Iqbal; J. Qadir; G. Tyson; I. Castro; A. Razi; M. N. K. Boulos; A. Weller; J. Crowcroft | Leveraging Data Science to Combat COVID-19: A Comprehensive Review | IEEE Transactions on Artificial Intelligence | 6 |
| 128 | IEEE | R. Minetto; M. P. Segundo; G. Rotich; S. Sarkar | Measuring Human and Economic Activity From Satellite Imagery to Support City-Scale Decision-Making During COVID-19 Pandemic | IEEE Transactions on Big Data | 6 |
| 129 | IEEE | J. R. Bhat; S. A. Alqahtani | 6G Ecosystem: Current Status and Future Perspective | IEEE Access | 6 |
| 130 | IEEE | Z. Ning; P. Dong; M. Wen; X. Wang; L. Guo; R. Y. K. Kwok; H. V. Poor | 5G-Enabled UAV-to-Community Offloading: Joint Trajectory Design and Task Scheduling | IEEE Journal on Selected Areas in Communications | 6 |
| 131 | IEEE | A. N. Navaz; M. A. Serhani; H. T. El Kassabi; N. Al-Qirim; H. Ismail | Trends, Technologies, and Key Challenges in Smart and Connected Healthcare | IEEE Access | 6 |
| 132 | IEEE | B. Benreguia; H. Moumen; M. A. Merzoug | Tracking COVID-19 by Tracking Infectious Trajectories | IEEE Access | 6 |
| 133 | IEEE | L. -J. Peng; X. -G. Shao; W. -M. Huang | Research on the Early-Warning Model of Network Public Opinion of Major Emergencies | IEEE Access | 6 |
| 134 | IEEE | G. Pang; G. Yang; Z. Pang | Review of Robot Skin: A Potential Enabler for Safe Collaboration, Immersive Teleoperation, and Affective Interaction of Future Collaborative Robots | IEEE Transactions on Medical Robotics and Bionics | 6 |
| 135 | IEEE | S. Miao; C. Shen; X. Feng; Q. Zhu; M. Shorfuzzaman; Z. Lv | Upper Limb Rehabilitation System for Stroke Survivors Based on Multi-Modal Sensors and Machine Learning | IEEE Access | 6 |
| 136 | IEEE | J. Sun; F. Khan; J. Li; M. D. Alshehri; R. Alturki; M. Wedyan | Mutual Authentication Scheme for the Device-to-Server Communication in the Internet of Medical Things | IEEE Internet of Things Journal | 6 |
| 137 | IEEE | C. T. Nguyen; Y. M. Saputra; N. V. Huynh; N. -T. Nguyen; T. V. Khoa; B. M. Tuan; D. N. Nguyen; D. T. Hoang; T. X. Vu; E. Dutkiewicz; S. Chatzinotas; B. Ottersten | A Comprehensive Survey of Enabling and Emerging Technologies for Social Distancing—Part I: Fundamentals and Enabling Technologies | IEEE Access | 6 |
| 138 | IEEE | J. Fang; H. Hou; L. Pan; Q. Deng; H. Pang; Y. Ye | Dynamic Planning Method for Drug Distribution in Earthquake Response Based on Sliding Time Window Series | IEEE Access | 6 |
| 139 | IEEE | E. F. Ohata; G. M. Bezerra; J. V. S. d. Chagas; A. V. Lira Neto; A. B. Albuquerque; V. H. C. d. Albuquerque; P. P. Reboucas Filho | Automatic detection of COVID-19 infection using chest X-ray images through transfer learning | IEEE/CAA Journal of Automatica Sinica | 6 |
| 140 | IEEE | C. Tang; S. Xia; M. Qian; B. Wang | Deep Learning-Based Vein Localization on Embedded System | IEEE Access | 6 |
| 141 | IEEE | L. Gao; Y. Liu; Z. Wang; Y. He; P. Wang; Y. Li; L. Li; X. Yu; J. Yu | High-Mechanical-Resolution Pressure Sensor Based on Melt-Blown Fibers in Integrated Wearable Mask for Respiratory Monitoring | IEEE Transactions on Electron Devices | 6 |
| 142 | IEEE | B. Wormuth; S. Wang; P. Dehghanian; M. Barati; A. Estebsari; T. P. Filomena; M. H. Kapourchali; M. A. Lejeune | Electric Power Grids Under High-Absenteeism Pandemics: History, Context, Response, and Opportunities | IEEE Access | 6 |
| 143 | IEEE | S. Miao; Y. Dang; Q. Zhu; S. Li; M. Shorfuzzaman; H. Lv | A Novel Approach for Upper Limb Functionality Assessment Based on Deep Learning and Multimodal Sensing Data | IEEE Access | 6 |
| 144 | IEEE | A. Hedayatipour; N. Mcfarlane | Wearables for the Next Pandemic | IEEE Access | 6 |
| 145 | IEEE | G. Yang; Z. Pang; M. Jamal Deen; M. Dong; Y. -T. Zhang; N. Lovell; A. M. Rahmani | Homecare Robotic Systems for Healthcare 4.0: Visions and Enabling Technologies | IEEE Journal of Biomedical and Health Informatics | 6 |
| 146 | IEEE | Y. Gao; H. Lin; Y. Chen; Y. Liu | Blockchain and SGX-Enabled Edge-Computing-Empowered Secure IoMT Data Analysis | IEEE Internet of Things Journal | 6 |
| 147 | IEEE | M. Irfan; H. Jawad; B. B. Felix; S. Farooq Abbasi; A. Nawaz; S. Akbarzadeh; M. Awais; L. Chen; T. Westerlund; W. Chen | Non-Wearable IoT-Based Smart Ambient Behavior Observation System | IEEE Sensors Journal | 6 |
| 148 | IEEE | Y. Qiu; H. Zhang; K. Long | Computation Offloading and Wireless Resource Management for Healthcare Monitoring in Fog-Computing-Based Internet of Medical Things | IEEE Internet of Things Journal | 6 |
| 149 | IEEE | C. Li; Y. Zhang; X. Li | Epidemic Threshold in Temporal Multiplex Networks With Individual Layer Preference | IEEE Transactions on Network Science and Engineering | 6 |
| 150 | IEEE | P. Zhu; J. Hu; Y. Zhang; X. Li | A Blockchain Based Solution for Medication Anti-Counterfeiting and Traceability | IEEE Access | 6 |
| 151 | IEEE | A. Angelucci; D. Kuller; A. Aliverti | A Home Telemedicine System for Continuous Respiratory Monitoring | IEEE Journal of Biomedical and Health Informatics | 6 |
| 152 | IEEE | D. Espindola; M. W. Wright | The Exponential Human – Social and Ethical Challenges in a Chaotic World | The Exponential Era: Strategies to Stay Ahead of the Curve in an Era of Chaotic Changes and Disruptive Forces | 6 |
| 153 | IEEE | D. Xu; T. Li; Y. Li; X. Su; S. Tarkoma; T. Jiang; J. Crowcroft; P. Hui | Edge Intelligence: Empowering Intelligence to the Edge of Network | Proceedings of the IEEE | 6 |
| 154 | IEEE | V. Chamola; V. Hassija; S. Gupta; A. Goyal; M. Guizani; B. Sikdar | Disaster and Pandemic Management Using Machine Learning: A Survey | IEEE Internet of Things Journal | 6 |
| 155 | IEEE | J. Indumathi; A. Shankar; M. R. Ghalib; J. Gitanjali; Q. Hua; Z. Wen; X. Qi | Block Chain Based Internet of Medical Things for Uninterrupted, Ubiquitous, User-Friendly, Unflappable, Unblemished, Unlimited Health Care Services (BC IoMT U<sup>6</sup> HCS) | IEEE Access | 6 |
| 156 | IEEE | Y. Qian; J. Shen; P. Vijayakumar; P. K. Sharma | Profile Matching for IoMT: A Verifiable Private Set Intersection Scheme | IEEE Journal of Biomedical and Health Informatics | 6 |
| 157 | IEEE | V. M. G. Abarca; P. R. Palos-Sanchez; E. Rus-Arias | Working in Virtual Teams: A Systematic Literature Review and a Bibliometric Analysis | IEEE Access | 6 |
| 158 | IEEE | S. Garg; N. S眉nderhauf; F. Dayoub; D. Morrison; A. Cosgun; G. Carneiro; Q. Wu; T. -J. Chin; I. Reid; S. Gould; P. Corke; M. Milford | Semantics for Robotic Mapping, Perception and Interaction: A Survey | Semantics for Robotic Mapping, Perception and Interaction: A Survey | 6 |
| 159 | IEEE | D. C. Nguyen; M. Ding; P. N. Pathirana; A. Seneviratne; J. Li; H. Vincent Poor | Federated Learning for Internet of Things: A Comprehensive Survey | IEEE Communications Surveys & Tutorials | 6 |
| 160 | IEEE | S. K. Zhou; H. Greenspan; C. Davatzikos; J. S. Duncan; B. Van Ginneken; A. Madabhushi; J. L. Prince; D. Rueckert; R. M. Summers | A Review of Deep Learning in Medical Imaging: Imaging Traits, Technology Trends, Case Studies With Progress Highlights, and Future Promises | Proceedings of the IEEE | 6 |
| 161 | IEEE | L. Chiaraviglio; A. Elzanaty; M. -S. Alouini | Health Risks Associated With 5G Exposure: A View From the Communications Engineering Perspective | IEEE Open Journal of the Communications Society | 6 |
| 162 | IEEE | M. H. A. Banna; T. Ghosh; M. J. A. Nahian; K. A. Taher; M. S. Kaiser; M. Mahmud; M. S. Hossain; K. Andersson | Attention-Based Bi-Directional Long-Short Term Memory Network for Earthquake Prediction | IEEE Access | 6 |
| 163 | IEEE | P. Siriaraya; Y. Wang; Y. Zhang; S. Wakamiya; P. Jeszenszky; Y. Kawai; A. Jatowt | Beyond the Shortest Route: A Survey on Quality-Aware Route Navigation for Pedestrians | IEEE Access | 6 |
| 164 | IEEE | D. Mar铆n-Suelves; S. L贸pez-G贸mez; M. M. Castro-Rodr铆guez; J. Rodr铆guez-Rodr铆guez | Digital Competence in Schools: A Bibliometric Study | IEEE Revista Iberoamericana de Tecnologias del Aprendizaje | 6 |
| 165 | IEEE | S. Landau | 5 CAN CONTACT-TRACING APPS BE EFFECTIVE TOOLS OF PUBLIC HEALTH? | People Count: Contact-Tracing Apps and Public Health | 6 |
| 166 | IEEE | M. A. Ferrag; O. Friha; L. Maglaras; H. Janicke; L. Shu | Federated Deep Learning for Cyber Security in the Internet of Things: Concepts, Applications, and Experimental Analysis | IEEE Access | 6 |
| 167 | IEEE | P. Leelaarporn; P. Wachiraphan; T. Kaewlee; T. Udsa; R. Chaisaen; T. Choksatchawathi; R. Laosirirat; P. Lakhan; P. Natnithikarat; K. Thanontip; W. Chen; S. C. Mukhopadhyay; T. Wilaiprasitporn | Sensor-Driven Achieving of Smart Living: A Review | IEEE Sensors Journal | 6 |
| 168 | IEEE | Y. E. Chan; R. Krishnamurthy; A. S. Ghawe | Information Technology Alignment and Innovation: 30 Years of Intersecting Research | Information Technology Alignment and Innovation: 30 Years of Intersecting Research | 6 |
| 169 | IEEE | O. A. Wahab; A. Mourad; H. Otrok; T. Taleb | Federated Machine Learning: Survey, Multi-Level Classification, Desirable Criteria and Future Directions in Communication and Networking Systems | IEEE Communications Surveys & Tutorials | 6 |
| 170 | IEEE | G. Markarian; A. Staniforth | Countermeasures for Aerial Drones | Countermeasures for Aerial Drones | 6 |
| 171 | IEEE | V. Verboeket; S. H. Khajavi; H. Krikke; M. Salmi; J. Holmstr枚m | Additive Manufacturing for Localized Medical Parts Production: A Case Study | IEEE Access | 6 |
| 172 | IEEE | M. A. D. Souza; H. P. Kuribayashi; P. A. Saraiva; F. D. S. Farias; N. L. Vijaykumar; C. R. L. Franc锚s; J. C. W. A. Costa | A Techno-Economic Framework for Installing Broadband Networks in Rural and Remote Areas | IEEE Access | 6 |
| 173 | IEEE | B. Kemp; B. Lovett | The Handbook of Next-Generation Emergency Services | The Handbook of Next-Generation Emergency Services | 6 |
| 174 | IEEE | H. K. Bharadwaj; A. Agarwal; V. Chamola; N. R. Lakkaniga; V. Hassija; M. Guizani; B. Sikdar | A Review on the Role of Machine Learning in Enabling IoT Based Healthcare Applications | IEEE Access | 6 |
| 175 | IEEE | C. Coursey | The Practitioner's Guide to Cellular IoT | The Practitioner's Guide to Cellular IoT | 6 |
| 176 | IEEE | K. Pahlavan | Understanding Communications Networks – for Emerging Cybernetics Applications Revised Edition | Understanding Communications Networks - for Emerging Cybernetics Applications Revised Edition | 6 |
| 177 | IEEE | L. Willems | Understanding the Impacts of Autonomous Vehicles in Logistics | The Digital Transformation of Logistics: Demystifying Impacts of the Fourth Industrial Revolution | 6 |
| 178 | IEEE | A. Musamih; K. Salah; R. Jayaraman; J. Arshad; M. Debe; Y. Al-Hammadi; S. Ellahham | A Blockchain-Based Approach for Drug Traceability in Healthcare Supply Chain | IEEE Access | 6 |
| 179 | IEEE | V. Gupta; S. Sachdeva; S. Bhalla | A Novel Deep Similarity Learning Approach to Electronic Health Records Data | IEEE Access | 6 |
| 180 | IEEE | N. S. Labib; M. R. Brust; G. Danoy; P. Bouvry | The Rise of Drones in Internet of Things: A Survey on the Evolution, Prospects and Challenges of Unmanned Aerial Vehicles | IEEE Access | 6 |
| 181 | IEEE | N. Jamil; A. N. Belkacem; S. Ouhbi; C. Guger | Cognitive and Affective Brain–Computer Interfaces for Improving Learning Strategies and Enhancing Student Capabilities: A Systematic Literature Review | IEEE Access | 6 |
| 182 | IEEE | J. Enriquez | POSTSCRIPT: GAME CHANGERS | Right/Wrong: How Technology Transforms Our Ethics | 6 |
| 183 | IEEE | J. Enriquez | Index | Right/Wrong: How Technology Transforms Our Ethics | 6 |
| 184 | IEEE | THz Global, La Canada, CA, USA | Microwave Pioneers: Kam Lau, “μWaves Meet Photons” | IEEE Journal of Microwaves | 6 |
| 185 | IEEE | M. P. Bhandari | 10 Case Study: Bashudaiva Kutumbakka – The Entire World is Our Home and all Living Beings are Our Relatives; Why We Need to Worry About Climate Change? With Reference to Pollution Problems in the Major Cities of India, Nepal, Bangladesh and Pakistan | Getting the Climate Science Facts Right: The Role of the IPCC | 6 |
| 186 | IEEE | MIT Press | Index | Uncertain Archives: Critical Keywords for Big Data | 6 |
| 187 | IEEE | School of Intelligent Systems Engineering, Sun Yat-sen University, Shenzhen, China; Department of Electrical and Electronic Engineering, The University of Hong Kong, Hong Kong; Department of Information Technology, Uppsala University, Uppsala, Sweden; School of Information Sciences & Engineering, Lanzhou University, Lanzhou, China; Instituto de Telecomunica莽玫es Federal University of Piau铆, Teresina, Brazil; Department of Computer and Information Security, Sejong University, Seoul, South Korea | Special Issue on “Toward Intelligent Internet of Medical Things and its COVID-19 Applications and Beyond” | IEEE Internet of Things Journal | 6 |
| 188 | IEEE | F. Wang | Parallel Healthcare: Robotic Medical and Health Process Automation for Secured and Smart Social Healthcares | IEEE Transactions on Computational Social Systems | 6 |
| 189 | IEEE | IEEE | Guest Editorial Enabling Technologies in Health Engineering and Informatics for the New Revolution of Healthcare 4.0 | IEEE Journal of Biomedical and Health Informatics | 6 |
| 190 | IEEE | J. Sarkis | Editorial | IEEE Engineering Management Review | 6 |
| 191 | CNKI | 李小会,刘青花,蔡宗仁,李泉峰,胡晓慧,牛芳 | 叙事护理模式在新型冠状病毒肺炎患者护理中的应用 | 甘肃科技 | 3 |
| 192 | CNKI | 杨志林,陈杰,杨晓清,向光明 | 武汉某方舱医院469例新型冠状病毒肺炎患者中医病机核心及证型特点分析 | 成都中医药大学学报 | 2 |
| 193 | CNKI | 赵向东,王帮德,高飞,张园,周璞,周冰,潘超,张萍,唐颖馨,唐洲平 | 新型冠状病毒肺炎疫情中可穿戴设备的应用示例与展望 | 高科技与产业化 | 6 |
| 194 | CNKI | 程辉,袁柏春,孙晖,周琼,刘小莉,傅新巧,王晶晶,张颖聪 | 新型冠状病毒肺炎疫情期间方舱医院医患共同体的构建及启示 | 中国社会医学杂志 | 8 |
| 195 | CNKI | 王畅,李文浩,王大虎,黄媛,王春兰,刘慧,林伟权 | 武汉市某方舱医院新冠肺炎患者心理健康状况调查 | 预防医学论坛 | 4 |
| 196 | CNKI | 侯杨清 | 新冠肺炎期间方舱医院护士头面部压疮的两种防护方案的效果观察 | 当代护士(中旬刊) | 3 |
| 197 | CNKI | 祝清清,殷琼,王晓芬,龙菊华 | 新冠肺炎疫情期间方舱医院批量收治患者的应急护理对策 | 当代护士(下旬刊) | 3 |
| 198 | CNKI | 罗照春 | 从SARS到新冠：中医防治作用非凡 | 家庭医学 | 2 |
| 199 | CNKI | 赵侠,严向炜 | 新型冠状病毒肺炎疫情期间的标准法规综述与分析 | 暖通空调 | 6 |
| 200 | CNKI | 陈中伟,张俊飞,龙官保,冯珂,马汉宁,杨立山,章军建,向准 | 新冠状肺炎防治工作中方舱医院医护人员突发意外情况救治策略探讨 | 现代医院 | 2 |
| 201 | CNKI | 张少强,李晓凤,裴丽敏,张丽红,邓芳隽,陈金红,黄博,袁嘉璐,宋爽,李卓威,杜武勋 | 983例武汉方舱医院轻型与普通型新型冠状病毒肺炎患者临床表现与诊疗特征研究 | 辽宁中医杂志 | 2 |
| 202 | CNKI | 李小红,张丽艳,陶品月,梁榕,莫园园,卢舒雨,陈林,黄惠桥 | 院感督导护士主导的监督机制在武汉方舱医院医务人员感染防控中的应用 | 现代医药卫生 | 7 |
| 203 | CNKI | 童春南,孙胜招,严莉,王晓蓉,史册,张载福 | 线上正念干预对新冠肺炎方舱医院护士焦虑抑郁情绪的影响 | 浙江医学教育 | 4 |
| 204 | CNKI | 韩亦姣  Han YJ | 突发公共卫生事件应对背景下智慧方舱模式初探  Study on Smart Shelter Hospital Mode in Response to Public Health Emergency (in Chinese) | 中国应急救援  China Emergency Rescue | 0 |
| 205 | CNKI | 滕飞 | 举国同心的抗疫精神 | 新长征 | 6 |
| 206 | CNKI | 伊同英,李玉芳 | 以罗森塔尔效应为基础的心理干预在方舱医院患者中的应用及效果分析 | 当代护士(中旬刊) | 3 |
| 207 | CNKI | 王曾妍,高兴莲 | 新冠肺炎方舱医院分诊前台工作流程的建立与实践 | 当代护士(下旬刊) | 3 |
| 208 | CNKI | 庄涛静,潘宇芬,张宏宇 | 基于PERMA模式的心理干预在方舱医院新型冠状病毒肺炎患者中的应用 | 当代护士(上旬刊) | 3 |
| 209 | CNKI | 张华玲,刘俊峰,赵靖,向准,张竞由,翟晓辉,王健  Zhang HL, Liu JF, Zhao J, Xiang Z, Zhang JY, Zhai XH, Wang J. | 关于方舱医院安全防控的工作思考  Thinking about the safety prevention and control in the module hospital (in Chinese) | 中国卫生质量管理  Chinese Health Quality Management | 0 |
| 210 | CNKI | 肖燕,武筱旋,曹李耘,王卫民,张智,冯丽,彭莹莹,熊巨洋 | 医务社工介入公共卫生应急服务的管理实践 | 中华医院管理杂志 | 6 |
| 211 | CNKI | 陈剑明,连博,陈腾飞,徐霄龙,郭玉红,刘清泉 | 从中医药对新冠肺炎的诊治现状看中医急危重症医学的发展策略 | 时珍国医国药 | 2 |
| 212 | CNKI | 张伯礼 | 中医抗疫的文化自信 | 中国科技奖励 | 6 |
| 213 | CNKI | 申玉玲,徐玉凤 | “新冠肺炎”和“方舱医院”英译研究 | 文化产业 | 8 |
| 214 | CNKI | 孙易娜,吕文亮,李昊,肖勇,杨旻,杨海军,高清华,杨忠奇,寿折星,胡家才,马永刚,罗正武,程冰洁,刘林,沈峰,张思依,刘之义,徐晓惠,赵政,张涵灵,龙喻,梅青青,史瑞雯,刘海根 | 清肺排毒汤治疗轻型/普通型新型冠状病毒肺炎295例多中心临床研究 | 中医杂志 | 2 |
| 215 | CNKI | 夏漫,刘义兰,詹昱新,王莹,喻姣花,熊莉娟,张玲,王婷,王双 | 方舱医院开舱紧急收治新型冠状病毒肺炎患者的护理管理 | 护理学杂志 | 3 |
| 216 | CNKI | 詹爱琴,安轶,杨宗树,杨秀琴,陈春丽,陈琛,左维泽,赵新芳,张惠荣 | 武汉东西湖方舱医院新冠肺炎患者654例临床特点分析 | 中国热带医学 | 2 |
| 217 | CNKI | 王晶晶,周琼,孙晖,袁柏春,龙洪波  Wang JJ, Zhou Q, Sun H, Yuan BC, Long HB. | 新冠肺炎疫情下武汉方舱医院实施协同管理机制实践探索  Fangcang shelter hospitals in Wuhan during the COVID-19 epidemic: practice of collaborative management (in Chinese) | 中国医疗管理科学  Chinese Journal of Medical Management Sciences | 0 |
| 218 | CNKI | 孙宽,李丽勤,朱文成,魏伟,彭小祥,张良 | 疫情下方舱医院患者自我管理 | 解放军医院管理杂志 | 8 |
| 219 | CNKI | 李加冕,敬仁芝,代小松,刘婷,庞娟,张新星,肖力 | 方舱医院轻型、普通型新型冠状病毒肺炎患者营养风险的调查研究 | 右江民族医学院学报 | 8 |
| 220 | CNKI | 余莎莎,肖辉,李汉民  Yu SS, Xiao H, Li HM. | 新型冠状病毒肺炎疫情防治中方舱医院信息化设计及实践  Design and Practice of Informatization of Mobile Cabin Hospitals in COVID-19 Epidemic Prevention and Control (in Chinese) | 医学信息学杂志  Journal of Medical Informatics | 0 |
| 221 | CNKI | 莫素莹,邓仙炳,张为,傅应昌,赖逸贵,李铮,陈永拉,梁浩斌,罗浓伟,范慧婕 | 方舱医院轻型新型冠状病毒肺炎患者的心理和睡眠现状分析及其影响因素的研究 | 临床护理杂志 | 4 |
| 222 | CNKI | 何进椅,陈隽,张孝莉,郑晓莉,姜玉林,李红 | 预防新型冠状病毒肺炎的医用防护口罩选择及佩戴建议 | 中华灾害救援医学 | 6 |
| 223 | CNKI | 肖伟,汤小亮,邱雅凡,耿旭初 | 安全城市疫情防控体系构建初探——以武汉市新冠肺炎疫情防控为例 | 新建筑 | 6 |
| 224 | CNKI | 李强 | 新冠肺炎疫情防控：亿万国人齐心战“疫” | 中国医院院长 | 6 |
| 225 | CNKI | 马刚,冯珂,马汉宁,马小斌,杨笑,梅斌 | 方舱医院新冠肺炎普通型患者临床和CT特征与住院时间关系的研究 | 中华全科医学 | 2 |
| 226 | CNKI | 郑艳玲,尹德卢,周何军,陈田木 | 新型冠状病毒肺炎患者隔离点建设与运行:武汉市基层实践 | 中国全科医学 | 6 |
| 227 | CNKI | 刘荔,周浩,林波荣,余娟 | 新型冠状病毒肺炎疫情下隔离医院室内环境安全实时监测与防控策略 | 科学通报 | 6 |
| 228 | CNKI | 张玉兰 | 防控新冠肺炎疫情中方舱医院的护理工作要点 | 当代护士(中旬刊) | 3 |
| 229 | CNKI | 于淑云,吴丹,侯世科 | 定点收治医院与方舱医院医护人员的不良反应比较 | 解放军医院管理杂志 | 4 |
| 230 | CNKI | 苏义武,肖辉 | 新型冠状病毒肺炎疫情下的医院信息化支撑 | 医学信息学杂志 | 6 |
| 231 | CNKI |  | 2020中国会展业十大热词 | 中国会展 | 6 |
| 232 | CNKI | 杨佳,彭端亮,刘于嵩,孙颖,陈康 | 方舱医院批量救治新型冠状病毒肺炎轻症患者的医学实验室质量管理探讨 | 检验医学与临床 | 5 |
| 233 | CNKI | 范志俊 | 战“疫”夫妻档 | 当代兵团 | 6 |
| 234 | CNKI | 余玮 | 国士张伯礼：做患者可以托付生命的人（上） | 国际人才交流 | 6 |
| 235 | CNKI | 孙易娜,吕文亮,李昊,肖勇,杨旻,杨海军,高清华,杨忠奇,寿折星,胡家才,马永刚,罗正武,程冰洁,刘林,沈峰,张思依,刘之义,徐晓惠,赵政,张涵灵,龙喻,梅青青,史瑞雯,刘海根 | 清肺排毒汤治疗新型冠状病毒肺炎295例多中心临床研究 | 中医杂志 | 2 |
| 236 | CNKI | 王秉阳 | 保卫武汉，中国方案，防控常态化 | 中国经济周刊 | 6 |
| 237 | CNKI | 周晓芬,钱欣,蔡菲莉,陈玉芳,薛贻敏,陈刚,何进椅,程思文,黄平辉,郑晓莉,吕少静,王炜维 | 武汉方舱医院新型冠状病毒肺炎隔离病房工作人员穿脱个体防护装备认知状况调查 | 中华实验和临床病毒学杂志 | 8 |
| 238 | CNKI | 方春妮,刘芳枝  Fang CN, Li FZ. | 新冠肺炎疫情危机下武汉方舱医院建设与体育场馆功能拓展的研究  Construction of Wuhan Makeshift Hospital and Functional Expansion of Sports Stadiums under COVID-19 Crisis (in Chinese) | 武汉体育学院学报  Journal of Wuhan Institute of Physical Education | 0 |
| 239 | CNKI | 李斌,张亚男,吕铁成,黄亮,毛轶,李严 | 高大空间建筑用作新冠肺炎方舱医院的通风空调改造探讨 | 暖通空调 | 7 |
| 240 | CNKI | 李坤,覃淋 | 构筑守护生命的铜墙铁壁 | 贵州日报 | 6 |
| 241 | CNKI | 郭潇雅 | 勇担抗疫使命 重塑医卫体系 | 中国医院院长 | 6 |
| 242 | CNKI | 李文 | “人民至上、生命至上”的中国答卷 | 中国卫生人才 | 6 |
| 243 | CNKI | 李荫龙,李卫松,邸铁涛,张敬杰 | 基于方舱医院的中医药用药模式探讨 | 贵州中医药大学学报 | 2 |
| 244 | CNKI | 赵艳妮,唐攀,李艳静,刘志伟 | 关于新型冠状病毒肺炎患者雾化吸入治疗的防护技巧 | 贵州中医药大学学报 | 2 |
| 245 | CNKI | 郑颜磊,胡荣华,张莉,李诗 | 武汉市方舱医院117例新型冠状病毒肺炎患者出院后随访结果 | 中华危重病急救医学 | 1 |
| 246 | CNKI | 夏锐,王阳,张锦 | 新型冠状病毒肺炎方舱医院压差控制的探讨 | 建筑热能通风空调 | 7 |
| 247 | CNKI | 陈骊,李青凌,池浩,李智 | 借鉴抗疫“方舱医院”经验 提升我军批量伤员收治能力 | 西南国防医药 | 8 |
| 248 | CNKI | 叶燕,张丽萍,何细飞 | 方舱医院在应对新型冠状病毒肺炎疫情中的运行模式 | 中国临床护理 | 7 |
| 249 | CNKI | 冯韬,杨灿华,李桂迎,梁道业,贾新菊,唐吉平,邹鑫森,李峥 | 方舱医院轻型新型冠状病毒肺炎患者931例临床特点与管理探讨 | 广东医学 | 2 |
| 250 | CNKI |  | 为武汉战疫贡献绿色力量——陕西森工医院援鄂医疗队抗疫事迹 | 绿色中国 | 6 |
| 251 | CNKI | 王佩,车云峰,林薇,杨陆 | 新冠肺炎疫情相关突发公共卫生事件词汇英译浅析 | 中国翻译 | 8 |
| 252 | CNKI | 杨道良,陈龙云,顾俊杰,陈亮亮,季海峰,陈玄玄 | 方舱医院新型冠状病毒肺炎患者心理健康状况及心理干预 | 中国健康心理学杂志 | 4 |
| 253 | CNKI | 余恒毅,任秀华,祁星星,左琴,刘东 | 阿比多尔、清肺排毒汤、连花清瘟胶囊、金叶败毒颗粒对某方舱医院轻型/普通型新冠肺炎患者疗效的回顾性研究 | 中药药理与临床 | 2 |
| 254 | CNKI | 王婷,刘丽萍,张芳兰,杜松 | 新冠肺炎疫情期间方舱医院管理的实践探讨 | 卫生职业教育 | 7 |
| 255 | CNKI | 刘于嵩,彭端亮,杨佳,陈敦雁,袁军,贾红兵,于思远,陈欢欢,黎颖,杨宗树,陈康 | 方舱医院检验科的建设 | 现代医药卫生 | 5 |
| 256 | CNKI | 王曾妍,高兴莲,崔宇杨,黄靖,杨千贺 | 新型冠状病毒肺炎轻症、普通型患者焦虑、抑郁状况分析 | 心理月刊 | 4 |
| 257 | CNKI | 万娟 | 方舱医院103例新型冠状病毒肺炎患者的中医护理 | 天津护理 | 3 |
| 258 | CNKI | 张再鹏,陈焰华,雷建平,张德银,赵彬,刘荔 | 某方舱医院的通风空调系统设计 | 暖通空调 | 7 |
| 259 | CNKI | 罗西贝,凌瑞杰,丁亚兴,王一颖  Luo XB, Ling RJ, Ding YX, Wang YY. | 新冠肺炎疫情下方舱医院的院感管理实践  Practice of Nosocomial Infection Management in Shelter Hospital under COVID-19 Pandemic Chinese (in Chinese) | 中国社会医学杂志  Chinese Journal of Social Medicine | 0 |
| 260 | CNKI | 张明鸣,李刚,戴炎杉,屈莉红,初曙光 | 方舱式应急CT在新型冠状病毒肺炎疫区使用的初步经验 | 中国医学计算机成像杂志 | 2 |
| 261 | CNKI | 唐思哲,邵建文,王锦帆 | 新冠肺炎轻症患者诊疗中医患沟通的作用及启示 | 中国医学伦理学 | 3 |
| 262 | CNKI | 王荣,刘春英,靳英辉,罗丽娇,曾淑豪,杨冰香,欧阳艳琼 | 新型冠状病毒肺炎流行下方舱医院护理团队的构建与管理策略 | 医学新知 | 3 |
| 263 | CNKI | 邹辉煌,葛高琪,秦国顺,胡玉娜,张红梅,李黎明 | 107例方舱医院新型冠状病毒肺炎患者创伤后成长现状及影响因素分析 | 护理学报 | 4 |
| 264 | CNKI | 赵洁,郑传胜,余建明,吴红英,雷子乔 | 方舱医院车载CT在新型冠状病毒肺炎检查中的防护问题及对策 | 中华放射医学与防护杂志 | 2 |
| 265 | CNKI | 童旭芳,历风元 | 武汉方舱医院新型冠状病毒肺炎患者92例诊治体会 | 江西医药 | 2 |
| 266 | CNKI | 李昀泽,姚阳婧,高坤,万凌峰,常诚,李建军 | 安神颗粒治疗新型冠状病毒肺炎合并失眠110例临床疗效观察 | 辽宁中医杂志 | 2 |
| 267 | CNKI | 金雪艳,杨春斌,莫兴邦,杨国明,余文娟 | 方舱医院新冠肺炎患者心理状态分析 | 卫生职业教育 | 4 |
| 268 | CNKI | 周国红,尹良爽,朱文礼,张成元 | 武汉方舱医院患者的心理状况及心理健康异常影响因素分析 | 安徽医学 | 4 |
| 269 | CNKI | 刘聚伟,卢恒志,徐敏,吴佳佳 | 军队医院构建医疗方舱单元抗击新冠肺炎疫情的探索 | 解放军医院管理杂志 | 2 |
| 270 | CNKI |  | 宏图大道加油站获全国抗疫先进表彰 | 中国石油企业 | 6 |
| 271 | CNKI | 刘英俊,彭云涌,温念姗,楚加庆,张明,谢敏,贺方圆,李莹莹 | 基于平疫结合的大型公共场馆改造模式探讨 | 新型建筑材料 | 7 |
| 272 | CNKI | 刘萍,伊同英,傅文婷,张君娜,范美玲,齐玉龙,李海鸿 | 新型冠状病毒肺炎疫情防控一线（方舱医院）护理人员压力源分析及应对策略 | 卫生职业教育 | 4 |
| 273 | CNKI |  | “很勇敢,去了很多危险的地方”——记全国抗击新冠肺炎疫情先进个人、澎湃新闻记者魏凡 | 新闻战线 | 6 |
| 274 | CNKI | 韩毅 | “危险在前,我亦在前”——记全国抗击新冠肺炎疫情先进个人、重庆日报记者谢智强 | 新闻战线 | 6 |
| 275 | CNKI | 赵梓涵 | “向险而行是记者本分”——记全国抗击新冠肺炎疫情先进个人、中国妇女报记者王长路 | 新闻战线 | 6 |
| 276 | CNKI | 钟文俊 | 用镜头记录抗疫现场 用画面定格感人瞬间——记全国抗击新冠肺炎疫情先进个人、湖北日报社摄影记者柯皓 | 新闻战线 | 6 |
| 277 | CNKI | 吴浩 | “我不是在逆行,只是朝着目标出发”——记全国抗击新冠肺炎疫情先进个人、四川日报记者李寰 | 新闻战线 | 6 |
| 278 | CNKI |  | 一场不辞不惧的逆行 见证历史记录希望——记全国抗击新冠肺炎疫情先进个人、重庆华龙网集团记者周盈 | 新闻战线 | 6 |
| 279 | CNKI | 陈芳,安蓓,白洁,屈婷,胡喆,侯文坤 | 同舟共济战“疫”记——中国抗击新冠肺炎疫情全纪实 | 台声 | 6 |
| 280 | CNKI | 周俊辉,孙慧敏,黄文莉 | 分时段日督查模式在武汉方舱医院护理质量管理中的应用 | 中国护理管理 | 3 |
| 281 | CNKI |  | 9月劳动榜样 | 中国工人 | 6 |
| 282 | CNKI | 刘珺,张向阳,邓略,张晓丽,张莉莉,贺青,曹婧  Liu J, Zhang XY, Deng L, Zhang XL, Zhang LL, He Q, Cao J | 信息化野战方舱医院在新冠肺炎疫情防控中的卫勤保障作用  The Effect of Health Service Support of the Informationized Mobile Field Hospital in the Prevention and Control of COVID-19 (in Chinese) | 中国数字医学  China Digital Medicine | 0 |
| 283 | CNKI | 本刊编辑部 | 以国之名,向抗疫英雄致敬! | 平安校园 | 6 |
| 284 | CNKI |  | 致敬抗疫英雄 国家级荣誉背后的故事 | 科学大观园 | 6 |
| 285 | CNKI | 陈文英 | 幸而有你，山河无恙！ | 科技创新与品牌 | 6 |
| 286 | CNKI | 董欣悦 | 在灾难中成长 | 中学生阅读(高中版)(上半月) | 6 |
| 287 | CNKI | 李昀泽,史锁芳,姚阳婧,陈安琪,曹欣然,李建军 | 新型冠状病毒肺炎恢复期患者86例临床特征分析 | 辽宁中医药大学学报 | 2 |
| 288 | CNKI | 罗照春,莫郑波 | 抗新冠肺炎中药方剂的科学性 | 家庭医学 | 2 |
| 289 | CNKI | 姚锋,孙丹妮 | 在大战大考中书写让人民满意的交通答卷 | 中国水运报 | 6 |
| 290 | CNKI | 湖北日报评论员 | 伟大抗疫精神生动诠释中国精神 | 湖北日报 | 6 |
| 291 | CNKI | 臧颖,田丽,王晓萍,刘颖琪,苏杰敏,刘静 | 新型冠状病毒肺炎疫情期间发热门诊方舱式扩建与管理 | 天津护理 | 6 |
| 292 | CNKI | 王芳,谢琥 | 洪山体育馆方舱医院改造设计解读 | 煤炭工程 | 7 |
| 293 | CNKI | 李昀泽,姚阳婧,裔楠,常荧荥,常诚,李建军 | 清肺饮治疗新型冠状病毒肺炎合并高血压病患者152例临床疗效分析 | 浙江中医药大学学报 | 2 |
| 294 | CNKI | 韩树丽 | Vlog+新闻在新冠肺炎疫情报道中的实践应用 | 青年记者 | 6 |
| 295 | CNKI | 任慧娟,雷憾,孙贵新,李慧,白建文,唐伦先 | 94例武汉方舱医院新型冠状病毒肺炎患者临床特点分析 | 中华卫生应急电子杂志 | 2 |
| 296 | CNKI | 杨永峰,葛宁海,李佳欣,汤苏川,欧阳春,陈旭峰 | 新型冠状病毒肺炎疫情中方舱医院的建设与思考 | 中华传染病杂志 | 7 |
| 297 | CNKI | 张硕,张磊,王树苓,封继宏,朱津丽,雒明池 | 中医治疗新型冠状病毒肺炎病案分析1例 | 天津中医药 | 2 |
| 298 | CNKI | 黄青,曾伟,蔡永辉  Huang Q, Zeng W, Cai YH. | 应对突发公共卫生事件的方舱医院运行管理标准化研究  Research on Operation and Management Standardization of Makeshift Hospital in Response to Public Health Emergency (in Chinese) | 中国标准化  China Standardization | 0 |
| 299 | CNKI | 王晶晶,孙晖,杨超 | 新冠肺炎疫情下方舱医护临时党支部实践与探索 | 学理论 | 8 |
| 300 | CNKI | 王荣,刘春英,蔡忠香,朱文芳,刘军,黄红丽 | 新冠肺炎疫情下方舱医院护士工作满意度与团队合作度现状及相关性分析 | 齐鲁护理杂志 | 4 |
| 301 | CNKI | 何隽,叶倩,黄礼明,周亚林,张敬杰 | 浅谈武汉方舱医院院感防控管理 | 贵州中医药大学学报 | 7 |
| 302 | CNKI | 雷琪慧 | 新冠肺炎疫情防控中临时党组织如何发挥战斗堡垒作用——以东西湖方舱医院为例 | 现代医院 | 8 |
| 303 | CNKI | 刘美,杨纯子,何细飞,陈凤菊,汪晖 | 新型冠状病毒肺炎疫情期方舱医护人员共情疲劳及影响因素分析 | 护理学杂志 | 6 |
| 304 | CNKI | 2 | 新型冠状病毒肺炎轻症患者发病早期临床诊治与发热的相关性 | 海南医学院学报 | 2 |
| 305 | CNKI | 罗西贝,凌瑞杰,丁亚兴,王一颖  Luo XB, Ling RJ, Ding YX, Wang YY. | 武汉江岸方舱医院医院感染预防与控制措施及效果评价  Evaluation of measures for prevention and control of health care associated infections in Wuhan Jiang’an Shelter Hospital during the outbreak of coronavirus disease-19 (in Chinese) | 中国病毒病杂志  Chinese Journal of Viral Diseases | 0 |
| 306 | CNKI | 张宇星 | 终端化生存 后疫情时代的城市升维 | 时代建筑 | 6 |
| 307 | CNKI | 徐玉兰,刘义兰,曹青,黄辉,曾娅,詹煜新,王莹,周慧敏,熊莉娟 | 新型冠状病毒肺炎疫情下方舱医院护理管理模式的构建 | 中华护理杂志 | 3 |
| 308 | CNKI | 邓海华 | 凝聚行业斗志 书写抗疫华章 | 中国报业 | 6 |
| 309 | CNKI | 黄晓颖,刘辉,李知非,刘晓亮,郭方凯,单春辉,赵明娟,暴云锋 | 方舱医院胸部CT检查流程与防控规范 | 中国医疗设备 | 2 |
| 310 | CNKI | 乔青翠,王蕊,李婷,李欣洁,倪立昆 | 79例湿热蕴肺证新型冠状病毒肺炎患者中医护理体会 | 天津中医药 | 2 |
| 311 | CNKI | 王莹,喻姣花,詹昱新,叶旭阳,徐玉兰,张艳,王亚玲 | 方舱医院护士护理新型冠状病毒肺炎患者体验的质性研究 | 护理学杂志 | 3 |
| 312 | CNKI |  | 武汉7位年过百岁新冠肺炎患者出院 | 课堂内外创新作文(高中版) | 6 |
| 313 | CNKI | 刘敏 | 方舱医院中国经验 | 中国医院院长 | 7 |
| 314 | CNKI | 祝益民,胡成平,唐凌志,邓志红,王文龙,李春辉,张玉,徐芙蓉,章迪,彭玥,孙倩莱,孙爽 | 新冠肺炎疫情防控国家建议书 | 中国医院院长 | 6 |
| 315 | CNKI | 叶舒婷,戴飞跃,胡华,徐寅,盛望,李妲,李菁,王军,彭杰,孙爽,林泉成,胡哲,龚后武,朱镇华,朱莹 | 方舱3号方配合八段锦治疗方舱医院113例2019冠状病毒病轻型和普通型患者临床疗效观察 | 中华中医药杂志 | 2 |
| 316 | CNKI | 汪起正 | “以舞战‘疫’”——兼谈舞蹈的身心自愈 | 当代舞蹈艺术研究 | 6 |
| 317 | CNKI | 胡利琳,朱锐,姚琳,杨玲 | 新型冠状病毒肺炎轻症患者临床特征分析 | 中国医药 | 2 |
| 318 | CNKI | 胡雪倩,马迪迪,刘国英,张淑华,王文艳 | 方舱医院新冠肺炎患者心理状况调查及护理对策 | 齐鲁护理杂志 | 4 |
| 319 | CNKI | 李环羽 | 抗疫英雄——李峥 | 蛇志 | 6 |
| 320 | CNKI | 李桂迎,谢馥鍪,冯韬,周方圆,陆建晶,潘佳楣,李峥 | 来自中国武汉的COVID-2019防疫经验:方舱医院的建设与管理 | 蛇志 | 7 |
| 321 | CNKI | 高枫,周相涵 | 基于新冠肺炎诊疗体系的建筑设计研究及实践 | 中国医院建筑与装备 | 7 |
| 322 | CNKI | 王曾妍,高兴莲,张进祥,高芳,张义丹 | 新型冠状病毒肺炎方舱医院感染控制的难点与对策 | 中国消毒学杂志 | 7 |
| 323 | CNKI | 叶丽莎,潘陶玲 | 武昌方舱医院新型冠状病毒感染的肺炎轻症患者护理管理体会 | 广西医学 | 3 |
| 324 | CNKI | 郭潇雅 | 马秀华：力保首都“南大门” | 中国医院院长 | 6 |
| 325 | CNKI |  | 他们是院士,也是战士 | 中学生阅读(初中版) | 6 |
| 326 | CNKI | 王烨,牛然,于欣平,赖建强 | 基于网络爬虫和膳食评估辅助技术对方舱医院新冠肺炎患者膳食结构与营养供应的分析 | 营养学报 | 8 |
| 327 | CNKI | 陈文玲,张瑾 | 中医药何以能在抗疫实践中大放异彩？ | 雷锋 | 6 |
| 328 | CNKI |  | 科技抗疫,共克时艰联影“四位一体”智能筛查分级防控解决方案 | 中国医疗设备 | 6 |
| 329 | CNKI | 窦月玲 | 疫情选题策划征稿·课堂革命(一) “战‘疫’情”哲学小课堂 | 中学政治教学参考 | 6 |
| 330 | CNKI | 陈爱民,杨挚,罗秋,柯文翔 | 书写劳动精神 奉献硬核力量——2020年湖北五一劳动奖状揭晓 | 湖北画报(上旬) | 6 |
| 331 | CNKI | 向准,黄河,杨春丽,冯珂,龙官保,梅斌,章军建,陈中伟 | 新型冠状病毒肺炎疫情下方舱医院医疗风险防控探索 | 中国医院管理 | 7 |
| 332 | CNKI | 刘俊峰,向准,赵靖,张竞由,张华玲,翟晓辉,王健 | 方舱医院在新型冠状病毒肺炎疫情防控中的重要意义 | 中国医院管理 | 7 |
| 333 | CNKI | 蒋莉君,杨潇,邱昌建,李进,张波,李涛 | 华西“阳光医院”实践应用于新型冠状病毒肺炎疫情下武汉方舱医院一线医务人员心理援助一例 | 中华精神科杂志 | 4 |
| 334 | CNKI | 刘建华 | 中医药战疫显身手 | 小康 | 6 |
| 335 | CNKI | 刘琦,张光银,刘学政,林靖宇,司天梅 | 轻型和普通型新冠肺炎患者焦虑症状的诊疗建议 | 精神医学杂志 | 4 |
| 336 | CNKI | 彭羽佳 | 新冠肺炎疫情下图书馆应急服务对策研究 | 江苏科技信息 | 6 |
| 337 | CNKI | 傅新巧,孙晖,辛艳姣,孙扬,徐小兵,舒琴,张义丹,杨超,李迪  Fu XQ, Sun H, Xin YJ, Sun Y, Xu XB, Shu Q, Zhang YD, Yang C, Li D. | 突发公共卫生事件应急管理中方舱医院医疗管理实践与思考——以江汉方舱医院为例  Practice and Thinking on Medical Management of Cabin Hospitals in Emergency Management of Public Health Emergency: Taking Jianghan Cabin Hospital as an Example (in Chinese) | 医学与社会  Medicine and Society | 0 |
| 338 | CNKI | 李文浩,黄媛,王春兰,王畅 | 方舱医院新型冠状病毒肺炎患者焦虑状况 | 中国健康心理学杂志 | 4 |
| 339 | CNKI | 倪扬,张莉华,姜文娴,金晶,彭美荣,苏亚霞,林嫚婷,刘巍,胡汉昆 | 武汉客厅方舱医院新型冠状病毒肺炎轻症患者药品使用情况分析 | 中国药师 | 2 |
| 340 | CNKI | Kent | 聚焦新冠肺炎 | 新世纪智能 | 6 |
| 341 | CNKI | Kent | 聚焦新冠肺炎 | 新世纪智能 | 1 |
| 342 | CNKI | 何细飞,刘清华,杨建国,王昭昭,张丽萍,叶燕,唐洲平,曾铁英 | 方舱医院新型冠状病毒感染患者睡眠质量现状及影响因素分析 | 中国临床护理 | 4 |
| 343 | CNKI | 田思维,李玲,何细飞,余洪兴,杨建国 | 新型冠状病毒肺炎疫情下方舱医院安全管理模式实践 | 临床口腔医学杂志 | 7 |
| 344 | CNKI | 何颖 | 从新冠肺炎疫情防控思考中国航天技术转民用 | 军民两用技术与产品 | 6 |
| 345 | CNKI | 苏义武,肖辉 | 医院信息化助力疫情防控 | 中国数字医学 | 6 |
| 346 | CNKI | 潘锋 | 方舱医院是应对突发公共卫生事件的强有力手段——访中国工程院院士、中国医学科学院北京协和医学院院校长王辰教授 | 中国医药导报 | 7 |
| 347 | CNKI | 张贝贝,李成伟,肖辉  Zhang BB, Li CW, Xiao H. | 新型冠状病毒肺炎疫情下方舱医院信息系统设计  Design of the Information System of the Cabin Hospital Under COVID-19 (in Chinese). | 中国数字医学  China Digital Medicine | 0 |
| 348 | CNKI | 华小黎,辜明,罗立,曾芳,张玉,史琛  Hua XL, Gu M, Luo L, Zeng F, Zhang Y, Shi C. | “零接触”信息化药学服务在医院防控新冠肺炎疫情中的应用  The Application of "Zero Contact" Informationized Pharmaceutical Service in the Prevention and Control of COVID-19 in Hospitals (in Chinese) | 中国数字医学  China Digital Medicine | 0 |
| 349 | CNKI | 郭潇雅,于珊珊 | 疫情中闪耀白求恩精神 | 中国医院院长 | 6 |
| 350 | CNKI | 陆真,宋燕波,王梦兰,王蕾,周婷婷 | 方舱医院新型冠状病毒肺炎患者对健康教育需求的调查分析 | 中西医结合护理(中英文) | 4 |
| 351 | CNKI | 詹昱新,喻姣花,刘义兰,王莹,夏漫,王培红,杨霞,徐玉兰,孙晖 | 新型冠状病毒肺炎防控中方舱医院护理应急管理的实践 | 解放军护理杂志 | 3 |
| 352 | CNKI | 风扬 | 武汉女孩的方舱日记,被爱温暖的人不害怕 | 伴侣 | 8 |
| 353 | CNKI | 唐元兢 | 在战“疫”大考中培养当代医学生的责任意识和担当精神 | 文教资料 | 6 |
| 354 | CNKI | 秦永菊,柴丹,王挺,文燕,莫慧,陈珑,何苗,林立宇 | 疫病背景下大型集体收治场所(方舱医院)开展中医药适宜技术的思考 | 实用中医内科杂志 | 2 |
| 355 | CNKI |  | 张伯礼：提出中医药进入方舱医院 | 雷锋 | 2 |
| 356 | CNKI | 刘婉琳,敬仁芝,郑柳涛,高义,李加冕,刘一弦 | 罗伊适应模式下广场舞行为在方舱医院轻型新型冠状病毒肺炎病人中应用理论基础探讨 | 全科护理 | 4 |
| 357 | CNKI | 侯敏,滕永林 | 词述中国战“疫” | 语言战略研究 | 6 |
| 358 | CNKI | 本刊编辑部,孙明华,王继勇,董雷,耿肃竹 | 云端大数据 | 创新世界周刊 | 6 |
| 359 | CNKI | 刘园,郑传胜,雷子乔,杨杨,许斯,吴继军,余建明,许晓泉,刘莉,吴红英 | 方舱医院新型冠状病毒肺炎CT检查的防控体会 | 中华放射学杂志 | 2 |
| 360 | CNKI | 何细飞,程捷,杨建国,周欣宇,李玲,唐洲平,曾铁英,汪晖 | 新型冠状病毒肺炎疫情下方舱医院护理管理实践 | 护理学杂志 | 3 |
| 361 | CNKI | 周君,杨勇,黄雷,陈康,张文超,熊伟  Zhou J, Yang Y, Huang L, Chen K, Zhang W, Xiong W. | 疫情期间“方舱医院”医药护协作医院感染防控模式实践与效果  Practice and Effect of Physician – Pharmacist - Nurse Cooperation Mode in the Prevention and Control of Nosocomial Infection in Fangcang Hospitals (in Chinese) | 中国药业  China Pharmaceuticals | 0 |
| 362 | CNKI | 王涵,顾中盛,李浩,孟玲,王永庆,魏继福 | 新型冠状病毒肺炎期间武汉方舱医院供应药品的特殊存储条件分析 | 中国药业 | 8 |
| 363 | CNKI | 张晨曦,杜宇,赵文嘉 | 战“疫”中坚 岐黄亮剑 记抗疫中医急危重症专家刘清泉 | 中国卫生人才 | 6 |
| 364 | CNKI | 李岩,王金梅 | 基于方舱医院条件下新冠肺炎患者高效护理策略 | 齐鲁护理杂志 | 3 |
| 365 | CNKI | 思齐 | 为爱妻加油，方舱医院走出“小品哥” | 劳动保障世界 | 6 |
| 366 | CNKI | 董善京,王茜娜,高崚,高希言 | 辨证施灸治疗方舱医院36例新型冠状病毒肺炎患者腹泻疗效观察 | 中国针灸 | 2 |
| 367 | CNKI | 梁锦军,丁文茂,李良长,李卫晖,熊娟,万军 | 方舱医院分级救治模式在新型冠状病毒肺炎防控中的实践 | 解放军医院管理杂志 | 2 |
| 368 | CNKI | 席新学,汪晖,毛靖,何细飞,刘美,余洪兴  Xi XX, Wang H, Mao J, He XF, Liu M, Yu HX. | 方舱医院中新型冠状病毒肺炎患者的安全管理  Difficulties and coping strategies of safety management for inpatients with coronavirus disease 2019 at cabin hospital | 中华护理杂志  Chinese Journal of Nursing | 0 |
| 369 | CNKI | 张学,张文瑾,王秀梅,牛彦斌,郭振山,郝铁旦 | 新型冠状病毒肺炎方舱医院感染防控管理实践 | 中华护理杂志 | 7 |
| 370 | CNKI | 李筠,戴遥,胡柳,陶红兵,刘静,何艳丽,李茜 | 新型冠状病毒肺炎疫情下方舱医院护理团队应急管理实践探析 | 中华护理杂志 | 3 |
| 371 | CNKI | 曾珠,邓永鸿,彭洁婧 | 方舱医院中新型冠状病毒肺炎患者的护理管理 | 中华护理杂志 | 3 |
| 372 | CNKI | 张伟,江海娇,鲁卫华,姜小敢,廖茂蕾,杨尚志,周全,颜浩 | 方舱医院新型冠状病毒肺炎患者心理干预及康复经验总结 | 中华护理杂志 | 4 |
| 373 | CNKI | 高兴莲,沈剑辉,王曾妍,黄靖,杨千贺,郑好,张玉司 | 方舱医院收治新型冠状病毒肺炎患者护理管理策略 | 中华护理杂志 | 3 |
| 374 | CNKI | 吴迎华,戴文,熊燕,刘锐,张驰浩 | 武汉方舱医院收治新型冠状病毒肺炎患者的护理管理及体会 | 中华护理杂志 | 3 |
| 375 | CNKI | 任秀华,祁星星,左琴,汤杰,刘东 | 方舱医院813例新型冠状病毒肺炎患者治疗用药分析 | 医药导报 | 2 |
| 376 | CNKI | 周其源,洪瑛,宛超  Zhou QY, Hong Y, Wan C. | 方舱医院给排水设计要点  Key Points of the Water Supply and Drainage Design for Mobile Cabin Hospital (in Chinese) | 华中建筑  Huazhong Architecture | 0 |
| 377 | CNKI |  | 全国城市管理执法系统全力以赴做好新冠肺炎疫情防控工作 | 四川建筑 | 6 |
| 378 | CNKI |  | 行业类媒体人共谱抗疫之歌 | 报林 | 6 |
| 379 | CNKI | 高雪屏,谭立文,王小平 | 抗疫日记之——有新冠肺炎患者突发“大闹方舱”？——记武昌洪山方舱首例精神科转诊个案 | 国际精神病学杂志 | 4 |
| 380 | CNKI | 潘锋 | 中医药深度介入新冠肺炎诊疗全过程——访中国工程院院士、中央指导组专家组成员张伯礼教授 | 中国医药导报 | 6 |
| 381 | CNKI | 唐利军,周文珊,刘盛,杨想军,霍细香,谢曙光,李静,刘安生,唐诗迪,雷子乔,陈为民,牛延涛 | 新型冠状病毒肺炎放射诊断检查场所的感染控制与辐射安全监测和分析 | 中华放射医学与防护杂志 | 2 |
| 382 | CNKI | 王欢,吴波,徐丽莹,徐海波 | 新型冠状病毒肺炎疫情期间方舱医院方舱CT影像检查流程及规范经验 | 中华放射医学与防护杂志 | 2 |
| 383 | CNKI | 林雪君,常四铁,焦文敏,谌兴鑫 | 新冠疫情下基于武汉市医废处置及应急管理的思考 | 环境工程 | 6 |
| 384 | CNKI | 李天红,田霞,陈思,李小璐,宋俪婵,杨柳 | 新型冠状病毒肺炎轻症患者集中收治方舱医院的分诊管理 | 护理学杂志 | 3 |
| 385 | CNKI | 余锦芬,宋玉凯,费菲,孙卫强,宋祖峰 | 基于机器学习和动力学模型的湖北省新型冠状病毒肺炎疫情分析 | 生物医学工程研究 | 6 |
| 386 | CNKI | 肖伟,宋奕 | 以快应变：新冠肺炎疫情下的“抗疫设计”思考 | 建筑学报 | 6 |
| 387 | CNKI |  | 群论：当代城市·新型人居·建筑设计 | 建筑学报 | 6 |
| 388 | CNKI | 龙灏,张程远 | 区域联动 战略储备 平战双轨——基于历史和现实超大规模疫情的当代传染病医院设计 | 建筑学报 | 6 |
| 389 | CNKI | 祝益民,胡成平,唐凌志,邓志红,王文龙,李春辉,张玉,徐芙蓉,章迪,彭玥,孙倩莱,孙爽 | 遏制新冠肺炎 中国实践出击 | 健康报 | 6 |
| 390 | CNKI |  | 弘扬可贵精神 坚定道路自信 | 经济日报 | 6 |
| 391 | CNKI | 王萍 | 张伯礼:“我把胆留在武汉了!” | 中国人大 | 6 |
| 392 | CNKI | 孙增坤,何裕民 | 超越干涉主义，医疗也需要考虑“疾病耐受性”——兼论中医药介入新冠肺炎救治的新思考 | 医学与哲学 | 2 |
| 393 | CNKI |  | 国家卫健委就疫情防控工作向中国互联网协会致感谢信 | 互联网天地 | 6 |
| 394 | CNKI | 白敏,刘献强,吴伟强,孙凯,黄旭,靳怀宗 | 武汉江岸方舱医院472例新型冠状病毒肺炎临床特征分析 | 临床荟萃 | 2 |
| 395 | CNKI | 徐建华 | 专业、坚守与扶持:公共图书馆应对新冠肺炎疫情的实践特征——“应对新冠肺炎疫情的公共图书馆实践与思考”专题导言 | 图书与情报 | 6 |
| 396 | CNKI | 金姬,刘绮黎 | 百日战疫 | 新民周刊 | 6 |
| 397 | CNKI | 姜浩峰 | 武汉解封！ | 新民周刊 | 6 |
| 398 | CNKI | 程金宝,王超,刘筠 | 移动方舱CT用于新型冠状病毒肺炎检查的工作流程与实践 | 国际医学放射学杂志 | 2 |
| 399 | CNKI | 傅菲,刘筠 | 方舱CT技术进展与临床应急使用现状 | 国际医学放射学杂志 | 2 |
| 400 | CNKI | 张天宝,姚璇,熊进峰,彭明军 | 新型冠状病毒肺炎方舱医院消毒与感染防控措施 | 中国消毒学杂志 | 7 |
| 401 | CNKI |  | 深圳市装饰行业协会会员企业同心助力援建医院 | 中国建筑装饰装修 | 6 |
| 402 | CNKI | 王晓红 | 中医药抗疫大显身手中医疗法深入人心 | 知识经济 | 6 |
| 403 | CNKI | 沈叶,赵振宇 | 你是我们眼中的英雄——全国人民抗击新冠肺炎疫情览述 | 中国纪检监察 | 6 |
| 404 | CNKI | 周洁 | 援鄂抗击新型冠状病毒肺炎多民族医护团队心理现状分析——以昆明医科大学第三附属医院为例 | 云南科技管理 | 4 |
| 405 | CNKI |  | 2020年3月谣言榜单 | 中国信息安全 | 6 |
| 406 | CNKI | 曹鑫彦,周欣宇,汪晖,向邱,刘晶晶,肖杨春 | 新型冠状病毒肺炎疫情防控中方舱医院的护理管理实践 | 中西医结合护理(中英文) | 3 |
| 407 | CNKI | 唐超 | 从SARS到COVID-19,在战“疫”中成长 | 中国医院院长 | 6 |
| 408 | CNKI | 白敏,卢振,任秋香,刘爱菊,刘旭,李超 | 方舱医院转院的新型冠状病毒肺炎患者57例临床特征分析 | 临床内科杂志 | 2 |
| 409 | CNKI | 李达,车志英 | 武汉方舱医院58例新冠肺炎患者中医证型分析 | 微量元素与健康研究 | 2 |
| 410 | CNKI | 林玲,李素云,娄湘红,刘茜,喻姣花,刘义兰,王培红,詹昱新,王莹 | 方舱医院轻症新型冠状病毒肺炎病人的护理与管理 | 护理研究 | 3 |
| 411 | CNKI | 向邱,何细飞,徐素琴,曹鑫彦,刘晶晶,肖扬春 | 方舱医院新型冠状病毒肺炎患者护理人文关怀实践研究 | 护理管理杂志 | 3 |
| 412 | CNKI | 陈顺达,汤杰,叶青,刘东  Chen SD, Tang J, Ye Q, Liu D. | 方舱医院无接触药品调剂模式实践  Practice and Discussion of Non-contact Drug Dispensing Pattern in Square Cabin Hospital (in Chinese) | 医药导报  Herald of Medicine | 0 |
| 413 | CNKI | 吴岩,刘志伟 | 战“疫”：全国科技界发出最强音 | 国际人才交流 | 6 |
| 414 | CNKI |  | 新型冠状病毒肺炎疫情期间方舱医院卫生防护指南 | 中华预防医学杂志 | 1 |
| 415 | CNKI | 苏玓,马黎,黄雷,陈康,王懿睿,张恩景,杨勇,孟敏  Su D, Ma L, Huang L, Chen K, Wang YR, Zhang EJ, Yang Y, Meng M. | 基于方舱医院药事管理对突发公共卫生事件药学服务体系建设的思考  Construction of Pharmaceutical Care System for Public Health Emergencies Based on the Pharmaceutical Administration in Wuhan Fangcang Hospitals (in Chinese) | 中国药业  China Pharmaceuticals | 0 |
| 416 | CNKI | 顾中盛,王涵,李浩,孟玲,王永庆,魏继福 | 新型冠状病毒肺炎疫情期间武汉方舱医院的供应药品分析 | 中国药业 | 2 |
| 417 | CNKI | 梁锦军,李十月,贺华,马永刚,丁文茂,万军 | 医疗集群组织模式在方舱医院中的实践与创新 | 中国医院管理 | 7 |
| 418 | CNKI | 张华玲,王健,翟晓辉,向准,赵靖,刘俊峰 | 新型冠状病毒肺炎疫情下方舱医院党建工作思考 | 中国医院管理 | 8 |
| 419 | CNKI |  | 中国-世界 | 中国报道 | 6 |
| 420 | CNKI | 陈东升,毛振华,毛宗福 | 武汉如何降低病死率 | 中国卫生 | 6 |
| 421 | CNKI | 宁艳阳 | 张伯礼：中医药防治是中国方案的亮点 | 中国卫生 | 6 |
| 422 | CNKI | 李菡丹 | 涂可蔼 是民警，也是新冠肺炎康复者 | 中华儿女 | 6 |
| 423 | CNKI | 徐飞 | 六大主题，“战疫”素材直击导写 | 全国优秀作文选(初中) | 6 |
| 424 | CNKI | 吴文娟,何丽华,刘波,王莉,雷撼,向准  Wu WJ, He LH, Liu B, Wang L, Lei H, Xiang Z. | 新冠肺炎疫情期间方舱医院院内感染管理探讨  Nosocomial infection control strategy in cabin hospitals during the epidemic of COVID-19 | 中华医院管理杂志  Chinese Journal of Hospital Administration | 0 |
| 425 | CNKI | 张义丹,丁宁,胡豫,孙晖,傅新巧,喻姣花,许栋,张明,张进祥  Zhang YD, Ding N, Hu Y, Sun H, Fu XQ, Yu JH, Xu D, Zhang M, Zhang JX. | 新冠肺炎疫情下武汉方舱医院运行管理模式及实践探析  Exploration and analysis of the management mode of a cabin hospital during the outbreak of COVID-19 (in Chinese) | 中华医院管理杂志  Chinese Journal of Hospital Administration | 0 |
| 426 | CNKI | 姚刚,张晓祥,汪火明,李金,田坚,王梁  Yao G, Zhang XX, Wang HM, Li J, Tian J, Wang L. | 新冠肺炎疫情期间方舱医院信息化建设实践与思考  Practice and thinking of the informationized cabin hospitals during COVID-19 epidemic (in Chinese) | 中华医院管理杂志  Chinese Journal of Hospital Administration | 0 |
| 427 | CNKI | 于思远,吴文娟 | COVID-19方舱医院医学检验科建设和管理初探 | 检验医学 | 5 |
| 428 | CNKI | 辛家东,王泽鹏,张法荣 | 中西医结合治疗新型冠状病毒肺炎概述 | 安徽中医药大学学报 | 2 |
| 429 | CNKI | 林晖,胡浩,李伟 | 科研攻关:高擎利剑驱疫魔 | 党员文摘 | 6 |
| 430 | CNKI |  | 国际 | 人民周刊 | 6 |
| 431 | CNKI | 喻姣花,孙晖,詹昱新,王莹,王培红,刘义兰,张义丹,傅新巧,熊莉娟 | 新型冠状病毒肺炎疫情防控中方舱医院的护理应急综合管理 | 护理学杂志 | 3 |
| 432 | CNKI | 江淀淀,温碧玲,叶丽莎,李惠颜,文彬,麦英姬,汤滢 | 新型冠状病毒肺炎疫情下的专职感控护士在方舱医院中的管理实践 | 广西医科大学学报 | 3 |
| 433 | CNKI | 本刊编辑部 | 同舟共济 共克时艰 | 建设科技 | 6 |
| 434 | CNKI | 梁浩,李宏军,李志勇,许远超,李宝山,刘岩,酒淼,张川,宫玮,龚维科  Liang H, Li HJ, Li ZY, Xu YC, Li BS, Liu Y, Jiu M, Zhang, C, Gong W, Gong WK. | 《工业建筑改造为方舱医院的建设运营技术指南（试行）》编制解读  Compilation Interpretation of “Technical Guidelines for Construction and Operation of the Industrial Buildings Renovated to Mobile Cabin Hospitals (Trial)” (in Chinese) | 建设科技  Construction science and technology | 0 |
| 435 | CNKI | 吴盛正,李柯研,彭成忠,叶瑞忠,李亚清,吕发勤 | 5G远程机器人超声评估方舱医院隔离病房新冠肺炎心肺功能1例 | 临床超声医学杂志 | 2 |
| 436 | CNKI | 叶林,周金,冯爱英 | 新型冠状病毒肺炎疫情期间方舱医院管理实践与思考 | 全科护理 | 7 |
| 437 | CNKI | 金姬 | 他们,超强求生欲! | 新民周刊 | 6 |
| 438 | CNKI | 果伟,贾菲,沙莎,臧彦楠,刘珊珊 | 方舱医院新型冠状病毒感染患者应激相关障碍的药物治疗刍议 | 药物不良反应杂志 | 2 |
| 439 | CNKI | 史锁芳,刘清泉 | 从“江夏方舱中医模式”探讨中医药在新型冠状病毒肺炎治疗中的价值 | 江苏中医药 | 2 |
| 440 | CNKI | 邹亮 | 新冠肺炎疫情下对城市规划的思考 | 中国建设报 | 6 |
| 441 | CNKI | 李鑫,乐磊,黄雷,毛孝容,刘婷,庞娟,李加冕,陈超华 | 基于新型冠状病毒肺炎疫情的方舱医院护理管理体系构建 | 实用医院临床杂志 | 3 |
| 442 | CNKI | 辜明,华小黎,陈骏,曾芳,周涛,张玉,史琛 | 江汉方舱医院药事管理与药学服务实践 | 中国药师 | 7 |
| 443 | CNKI | 肖伟,宋奕 | 新冠肺炎疫情下的城市“抗疫设计”反思 | 建筑技艺 | 6 |
| 444 | CNKI | 李雪莹,唐振英 | 人文关怀护理在武汉方舱医院新冠肺炎轻症患者中的应用 | 齐鲁护理杂志 | 3 |
| 445 | CNKI | 刘琼 | 从新冠肺炎疫情Vlog看新媒体时代新闻传播的新形式 | 声屏世界 | 6 |
| 446 | CNKI | 陈进宏,杨敏婕,卢绮萍 | 新型冠状病毒肺炎患者方舱医院集中收治模式下外科医师的作用 | 中华消化外科杂志 | 2 |
| 447 | CNKI | 孙润康,张晓祥,徐进,任宇飞,唐颖馨,潘超,张萍,杨卫东,张燕婷,唐洲平 | 基于5G技术的COVID-19方舱医院网络基础设施快速部署和应用探索 | 神经损伤与功能重建 | 7 |
| 448 | CNKI | 程芳,李强,曾芳,王冬园,韩勇,吕永宁,张玉 | 方舱医院290名新型冠状病毒肺炎患者用药现状分析与建议 | 中国医院药学杂志 | 2 |
| 449 | CNKI | 高枫 | 基于新冠肺炎诊疗体系的建筑设计研究 | 时代建筑 | 6 |
| 450 | CNKI |  | 焦点 | 中国医院建筑与装备 | 6 |
| 451 | CNKI | 辛丹 | 新冠肺炎疫情中对健康保险的思考 | 中国保险 | 6 |
| 452 | CNKI |  | 好消息 | 中国医院院长 | 6 |
| 453 | CNKI |  | 社会热词 | 今日中学生 | 6 |
| 454 | CNKI | 廖君,黎昌政 | 湖北全省确诊病例中医药使用率达88.9% | 中医药管理杂志 | 6 |
| 455 | CNKI | 刘玉林,陈长春,柯贤柱,梁新军,王建意,张俊,桂阳瑞雪,陈锋 | 新型冠状病毒肺炎防控中方舱CT应用及影像流程设计 | 临床放射学杂志 | 2 |
| 456 | CNKI | 龚卫静,周涛,徐晨枫,徐佳强,刘易慧,韩勇,曾芳,张玉  Gong WJ, Zhou T, Xu CF, Xu JQ, Liu YH, Han Y, Zeng, F, Zhang, Y. | 方舱医院线上药学服务模式的实践与探讨  The practice and discussion of online pharmaceutical service mode in mobile cabin hospital (in Chinese) | 中国医院药学杂志  Chinese Journal of Hospital Pharmacy | 0 |
| 457 | CNKI | 龚卫静,伍三兰,吴婷婷,曾芳,史琛,韩勇,吕永宁,张玉 | 方舱医院新型冠状病毒肺炎患者治疗的药学监护 | 医药导报 | 2 |
| 458 | CNKI | 徐霞 | 太极拳对方舱医院新型冠状病毒肺炎病人抑郁情绪及希望水平的影响 | 全科护理 | 4 |
| 459 | CNKI |  | 学会动态 | 科技传播 | 6 |
| 460 | CNKI | 薛睿杰 | 刘清泉：战“疫”中坚 岐黄亮剑——武汉江夏方舱医院院长、第一位奔赴抗疫一线的三甲中医院院长 | 北京人大 | 6 |
| 461 | CNKI |  | 学会动态 | 新媒体研究 | 1 |
| 462 | CNKI | 靳惠怡 | 中国建材联合会要求出台两项协会标准以抗击新冠肺炎疫情 | 中国建材 | 6 |
| 463 | CNKI | 田悦 | 共同战“疫”终将胜利 | 国际人才交流 | 6 |
| 464 | CNKI | 吴轩 | 患者教育怎么做 床头码里有文章 | 国际人才交流 | 6 |
| 465 | CNKI | 杨丰文,黄明,张俊华 | 应对疫情中医药救治有哪些优势——张伯礼院士权威解答 | 天津中医药 | 6 |
| 466 | CNKI | 危莉,胡豫,张义丹,张明,张进祥 | 新型冠状病毒肺炎疫情时期方舱医院运行实践与思考 | 中国医院管理 | 7 |
| 467 | CNKI | 王一颖,丁亚兴,罗西贝,朱思伟 | 新型冠状病毒肺炎疫情下武汉方舱医院的医院感染风险及对策 | 中国医院管理 | 7 |
| 468 | CNKI | 傅新巧,胡豫,孙晖,喻姣花,熊莉娟,鲁晓化,张义丹,李迪,舒琴,徐小兵,吕斌 | 新型冠状病毒肺炎疫情下武汉方舱医院运行机制及关键环节探析 | 中国医院管理 | 7 |
| 469 | CNKI | 单杰,张晓祥,李力,李金,王延昭,汪火明 | 基于信息化的方舱医院新型冠状病毒肺炎患者收治 | 中国医院管理 | 7 |
| 470 | CNKI |  | 战“疫”一线的瞬间 | 中国报道 | 6 |
| 471 | CNKI | 本刊编辑部 | 疫情回顾 | 源流 | 6 |
| 472 | CNKI |  | 传媒万象 | 电视研究 | 6 |
| 473 | CNKI | 王基德 | 14亿人一条心 抗“疫”，中国必胜！ | 青岛画报 | 6 |
| 474 | CNKI | 本刊记者 | 甲光向日金鳞开 | 清风 | 6 |
| 475 | CNKI | 杨丽,蒋丽,白阳,蒋磊,章述军,刘煜亮 | “方舱医院”条件下新型冠状病毒肺炎疑似或轻型患者的高效诊疗流程 | 第三军医大学学报 | 2 |
| 476 | CNKI | 唐景莉,袁芳 | 战“疫”中的高校力量 | 中国高等教育 | 6 |
| 477 | CNKI | 刘俊峰,翟晓辉,向准,赵靖,张竞由,王健 | 应对新型冠状病毒肺炎疫情的方舱医院建设管理探讨 | 中国医院管理 | 7 |
| 478 | CNKI |  | 中国纺织工业联合会流通分会:急速驰援，全力支持武汉方舱医院物资筹备 | 纺织服装周刊 | 6 |
| 479 | CNKI | 江志伟,马朝群,汪悦,周惠芳,朱佳,陈晓虎,陈玉根,吴文忠,沈历宗,周贤梅,王中秋,乔飞,汤忠华,周恩超,张彪,冯瑶,段倍蓓,戴正香,翟玉祥,方祝元 | 大疫之下，三甲中医院紧急响应机制的思与探 | 中国医院院长 | 6 |
| 480 | CNKI |  | 湖北武汉所有方舱医院全部休舱 | 人民周刊 | 6 |
| 481 | CNKI |  | 中国战“疫”进行时 | 中国经济周刊 | 6 |
| 482 | CNKI | 张贝贝,肖辉,李成伟,李志强 | 新型冠状病毒肺炎防治工作中方舱医院信息化快速支撑方案探讨 | 中国医院管理 | 7 |
| 483 | CNKI | 姜浩峰 | 向新冠肺炎疫情发起总攻！ | 新民周刊 | 6 |
| 484 | CNKI | 吴林 | 中国电信5G技术助力新冠肺炎患者远程诊疗 | 中国设备工程 | 6 |
| 485 | CNKI | 陈轸 | 防控复工 两手抓两手都要硬——专访中设协建筑设计分会副会长兼秘书长陈轸 | 建筑设计管理 | 6 |
| 486 | CNKI | 本刊综合 | 众志成城迎挑战 全力以赴战疫情 | 四川党的建设 | 6 |
| 487 | CNKI | 王凯军,常丽春,杨美娟,刘敏 | 从非典到新冠肺炎疫情我国医疗污水疫情三级防护体系建设与思考 | 给水排水 | 6 |
| 488 | CNKI | 姜浩峰 | 抗疫之战，进入决战时刻 | 新民周刊 | 6 |
| 489 | CNKI | 刘士永 | 管窥新冠肺炎疫情下的历史片段 | 中国科学报 | 6 |
| 490 | CNKI | 张伯礼 | 中医药在新冠肺炎疫情防治中发挥了哪些作用 | 学习时报 | 2 |
| 491 | CNKI | 杨勇,朱玉莲,廉江平,刘巍,苏玓,程志军,黄国鑫,马建龙,蒙龙,郭亚可,顾中盛,徐建国,吴慧敏,段婉晴,童荣生 | 方舱医院药事管理与药学服务模式探讨 | 医药导报 | 2 |
| 492 | CNKI | 陈进宏,杨敏婕,卢绮萍 | 新型冠状病毒肺炎患者方舱医院集中收治模式下外科医师的作用 | 中华消化外科杂志 | 1 |
| 493 | CNKI | 王卓 | 向奋战在武汉抗击新冠肺炎前线的临床药师队伍致以崇高敬意 | 药学服务与研究 | 6 |
| 494 | CNKI |  | 抗击新冠疫情时间线梳理 | 中国总会计师 | 6 |
| 495 | CNKI |  | 信息通信行业持续做好新冠肺炎疫情防控通信保障 | 电信工程技术与标准化 | 6 |
| 496 | CNKI | 刘红伟 | 战“疫”中的勘察设计力量 | 中国勘察设计 | 6 |
| 497 | CNKI | 彭碧波,郑静晨 | 新冠肺炎疫情防控的应急医疗经验 | 中国应急管理 | 6 |
| 498 | CNKI |  | 中国电信5G技术助力首例新冠肺炎患者远程超声诊疗成功实施 | 中国有线电视 | 6 |
| 499 | CNKI | 郭潇雅 | 白衣天使演绎最美逆行 | 中国医院院长 | 6 |
| 500 | CNKI |  | 关于新冠肺炎的一切 | 党的生活(黑龙江) | 6 |
| 501 | CNKI | 薛继军,朱强,李小奇,陈岩 | 新型冠状病毒肺炎疫情中方舱医院医疗运行模式探索 | 甘肃医药 | 7 |
| 502 | CNKI | 李建广,冯慧文,皇甫凌雨 | 战疫时间线 | 科学大观园 | 2 |
| 503 | CNKI |  | 热点 | 江淮法治 | 2 |
| 504 | CNKI |  | 武汉首个以中医为主的方舱医院开舱 | 台声 | 8 |
| 505 | CNKI |  | 图说国事 | 祖国 | 6 |
| 506 | CNKI | 田悦 | 同心协力 共克时艰 | 国际人才交流 | 6 |
| 507 | CNKI | 张妮,董长喜,张健 | 中医对新冠肺炎疗效有多大 | 环球时报 | 6 |

*Note: 0 represents that the literature is included in this review; 1 represents that the record is excluded because it is a duplicate record; 2 represents that the record is excluded because its research topic is the clinical characteristics, diagnosis, and treatment of COVID-19 patients; 3 represents that the record is excluded because its research topic is the nursing for patients; 4 represents that the record is excluded because its research topic is the mental health of patients and medical staffs; 5 represents that the record is excluded because its research topic is the construction of laboratories; 6 represents that the record is excluded because its research topic is not related to mobile cabin hospitals in China; 7 represents that the literature is excluded due to poor literature quality or no DOI number; 8 represents that the literature is excluded due to other reasons.

Supplementary Table 3. Sources and research contents of included literature.

| Source | References | Research contents |
| --- | --- | --- |
| Official website | Science, technology and Industrialization Center of the Ministry of Housing and Urban-Rural Development of the People's Republic of China. Notice on Issuing the “Technical Guidelines for Construction and Operation of the Industrial Buildings Renovated to Mobile Cabin Hospitals (Trial)” (2020). http://www.chinahvac.com.cn/Article/Index/6035 [Accessed February 25, 2020]. | Preparedness of mobile cabin hospitals   - Site selection - Conversion of public venues into hospital - Architectural layout |
| Official website | Housing and Urban-Rural Development Department of Hubei Province, China. Technical requirements for the Design and Conversion of Makeshift (FangCang) Hospitals (Revised Edition) (2020). https://zjt.hubei.gov.cn/zfxxgk/zc/zcjd/202004/t20200414_2222063.shtml [Accessed March 30, 2020]. | Preparedness of mobile cabin hospitals   - Site selection - Conversion of public venues into hospital - Architectural layout |
| Official website | Housing and Urban-Rural Development Department of Shandong Province, China. Guidelines for the design of shelter temporary emergency medical facilities (2020). http://zjt.shandong.gov.cn/art/2020/12/28/art_103756_10222432.html [Accessed December 28, 2020]. | Preparedness of mobile cabin hospitals   - Site selection - Conversion of public venues into hospital - Architectural layout |
| Official website | Housing and Urban-Rural Development Department of Zhejiang Province, China. Technical guidelines for shelter temporary hospitals (2020). http://jst.zj.gov.cn/art/2020/2/13/art_1229159347_48452710.html [Accessed February 13, 2020]. | Preparedness of mobile cabin hospitals   - Site selection - Conversion of public venues into hospital - Architectural layout |
| Official website | Housing and Urban-Rural Development Department of Shandong Province, China. Design guideline for emergency transformation of gymnasium into temporary medical center (2020). http://jsszfhcxjst.jiangsu.gov.cn/art/2020/2/27/art_8639_8987348.html [Accessed February 27, 2020]. | Preparedness of mobile cabin hospitals   - Site selection - Conversion of public venues into hospital - Architectural layout |
| Official website | The National Health Commission of the People's Republic of China. Manual for working in Fangcang shelter hospitals, 3rd edition (2020). https://mp.weixin.qq.com/s/va9vs4HuP8wRQM5fALQcrg [Accessed Febure 22, 2020]. | Preparedness of mobile cabin hospitals   - Functional zoning   Management of mobile cabin hospitals   - Management of nosocomial infections |
| Pubmed | Jiang H, Song P, Wang S, Yin S, Yin J, Zhu C, et al. Quantitative assessment of the effectiveness of joint measures led by Fangcang shelter hospitals in response to COVID-19 epidemic in Wuhan, China. BMC Infect Dis. (2021) 21:626. doi: 10.1186/s12879-021-06165-w | Effect and significance of mobile cabin hospital during the COVID-19 pandemic |
| Pubmed | He Q, Xiao H, Li HM, Zhang BB, Li CW, Yuan FJ, et al. Practice in Information Technology Support for Fangcang Shelter Hospital during COVID-19 Epidemic in Wuhan, China. J Med Syst. (2021) 45:42. doi: 10.1007/s10916-021-01721-y | Management of mobile cabin hospitals   - Information technology support |
| Pubmed | Zhang Y, Shi L, Cao Y, Chen H, Wang X, Sun G. Wuhan mobile cabin hospital: A critical health policy at a critical time in China. Medicine. (2021) 100:e24077. doi: 10.1097/md.0000000000024077 | Effect and significance of mobile cabin hospital during the COVID-19 pandemic |
| Pubmed | Liu P, Zhang H, Long X, Wang W, Zhan D, Meng X, et al. Management of COVID-19 patients in Fangcang shelter hospital: clinical practice and effectiveness analysis. Clin Respir J. (2021) 15:280-286. doi: 10.1111/crj.13293 | Preparedness of mobile cabin hospitals   - Architectural layout - Functional zoning - Management of mobile cabin hospitals - Information technology support |
| Pubmed | Zhang M, Wang L, Yu S, Sun G, Lei H, Wu W. Status of occupational protection in the COVID-19 Fangcang Shelter Hospital in Wuhan, China. Emerg Microbes Infect. (2020) 9:1835-1842. doi: 10.1080/22221751.2020.1803145 | Management of mobile cabin hospitals   - Management of nosocomial infections |
| Pubmed | Zhang D, Ling H, Huang X, Li J, Li W, Yi C, et al. Potential spreading risks and disinfection challenges of medical wastewater by the presence of Severe Acute Respiratory Syndrome Coronavirus 2 (SARS-CoV-2) viral RNA in septic tanks of Fangcang Hospital. Sci Total Environ. (2020) 741:140445. doi: 10.1016/j.scitotenv.2020.140445 | Management of mobile cabin hospitals   - Management of nosocomial infections |
| Pubmed | Zhou B, Wu Q, Zhao X, Zhang W, Wu W, Guo Z. Construction of 5G all-wireless network and information system for cabin hospitals. J Am Med Inform Assoc. (2020) 27:934-938. doi:10.1093/jamia/ocaa045 | Management of mobile cabin hospitals   - Information technology support |
| Pubmed | Shang L, Xu J, Cao B. Fangcang shelter hospitals in COVID-19 pandemic: the practice and its significance. Clin Microbiol Infect. (2020) 26:976-978. doi: 10.1016/j.cmi.2020.04.038 | Effect and significance of mobile cabin hospital during the COVID-19 pandemic |
| Pubmed | Yuan Y, Qiu T, Wang T, Zhou J, Ma Y, Liu X, et al. The application of Temporary Ark Hospitals in controlling COVID-19 spread: The experiences of one Temporary Ark Hospital, Wuhan, China. J Med Virol. (2020) 92:2019-2026. doi: 10.1002/jmv.25947 | Preparedness of mobile cabin hospitals   - Architectural layout - Functional zoning   Management of mobile cabin hospitals   - Management of nosocomial infections - Information technology support |
| Pubmed | Zhou F, Gao X, Li M, Zhang Y. Shelter Hospital: Glimmers of Hope in Treating Coronavirus 2019. Disaster Med Public Health Prep. (2020) 14:e3-e4. doi: 10.1017/dmp.2020.105 | Preparedness of mobile cabin hospitals   - Functional zoning |
| Pubmed | The COVID-19 Emergency Response Key Places Protection and Disinfection Technology Team, Chinese Center for Disease Control and Prevention. Health protection guideline of mobile cabin hospitals during COVID-19 outbreak (in Chinese). Zhonghua Yu Fang Yi Xue Za Zhi. (2020) 54:357-359. doi: 10.3760/cma.j.cn112150-20200217-00121 | Management of mobile cabin hospitals   - Management of nosocomial infections |
| Pubmed | Meng L, Qiu F, Sun S. Providing pharmacy services at cabin hospitals at the coronavirus epicenter in China. Int J Clin Pharm. (2020) 42:305-308. doi:10.1007/s11096-020-01020-5 | Management of mobile cabin hospitals   - Information technology support |
| Pubmed | Chen Z, He S, Li F, Yin J, Chen X. Mobile field hospitals, an effective way of dealing with COVID-19 in China: sharing our experience. Biosci Trends. (2020) 14:212-214. doi: 10.5582/bst.2020.01110 | Management of mobile cabin hospitals   - Organizational management |
| Pubmed | Yang Y, Wang H, Chen K, Zhou J, Deng S, Wang Y. Shelter hospital mode: How do we prevent COVID-19 hospital-acquired infection? Infect Control Hosp Epidemiol. (2020) 41:872-3. doi: 10.1017/ice.2020.97 | Management of mobile cabin hospitals   - Management of nosocomial infections |
| IEEE | Ding X, Clifton D, Ji N, Lovell NH, Bonato P, Chen W, et al. Wearable sensing and telehealth technology with potential applications in the coronavirus pandemic. IEEE Rev Biomed Eng. (2020) 14:48-70. doi: 10.1109/RBME.2020.2992838 | Management of mobile cabin hospitals   - Information technology support |
| IEEE | Marinelli M. Emergency healthcare facilities: managing design in a post Covid-19 world. IEEE Eng Manage Rev. (2020) 48:65-71. doi: 10.1109/EMR.2020.3029850 | Preparedness of mobile cabin hospitals   - Conversion of public venues into hospital |
| CNKI | 韩亦姣.突发公共卫生事件应对背景下智慧方舱模式初探[J].中国应急救援,2021(03):40-44.  Han YJ. Study on Smart Shelter Hospital Mode in Response to Public Health Emergency (in Chinese). China Emergency Rescue. (2021) 3:40-44. doi:10.19384/j.cnki.cn11-5524/p.2021.03.009 | Management of mobile cabin hospitals   - Information technology support |
| CNKI | 张华玲,刘俊峰,赵靖,向准,张竞由,翟晓辉,王健.关于方舱医院安全防控的工作思考[J].中国卫生质量管理,2021,28(05):40-43.  Zhang HL, Liu JF, Zhao J, Xiang Z, Zhang JY, Zhai XH, et al. Thinking about the safety prevention and control in the module hospital (in Chinese). Chin Health Qual Manage. (2021) 28(5):40-43. doi: 10.13912/j.cnki.chqm.2021.28.5.12 | Management of mobile cabin hospitals   - Organizational management - Management of nosocomial infections |
| CNKI | 王晶晶,周琼,孙晖,袁柏春,龙洪波.新冠肺炎疫情下武汉方舱医院实施协同管理机制实践探索[J].中国医疗管理科学,2021,11(02):50-53.  Wang JJ, Zhou Q, Sun H, Yuan BC, Long HB. Fangcang shelter hospitals in Wuhan during the COVID-19 epidemic: practice of collaborative management (in Chinese). Chin J Med Mgt Sci. (2021) 11:50-53. doi: 10.3969/j.issn.2095-7432.2021.02.010 | Management of mobile cabin hospitals   - Organizational management |
| CNKI | 余莎莎,肖辉,李汉民.新型冠状病毒肺炎疫情防治中方舱医院信息化设计及实践[J].医学信息学杂志,2021,42(02):66-69.  Yu SS, Xiao H, Li HM. Design and Practice of Informatization of Mobile Cabin Hospitals in COVID-19 Epidemic Prevention and Control (in Chinese). J Med Inf. (2021) 42:66-69. doi: 10.3969/j.issn.1673-6036.2021.02.014 | Management of mobile cabin hospitals   - Information technology support |
| CNKI | 方春妮,刘芳枝.新冠肺炎疫情危机下武汉方舱医院建设与体育场馆功能拓展的研究[J].武汉体育学院学报,2020,54(12):5-11.  Fang CN, Liu FZ. Construction of Wuhan Makeshift Hospital and Functional Expansion of Sports Stadiums under COVID-19 Crisis (in Chinese). J Wuhan Inst Phys Edu. (2020) 54:5-11. doi:10.15930/j.cnki.wtxb.2020.12.001 | Effect and significance of mobile cabin hospital during the COVID-19 pandemic |
| CNKI | 罗西贝,凌瑞杰,丁亚兴,王一颖.新冠肺炎疫情下方舱医院的院感管理实践[J].中国社会医学杂志,2020,37(05):465-467.  Luo XB, Ling RJ, Ding YX, Wang YY. Practice of Nosocomial Infection Management in Shelter Hospital under COVID-19 Pandemic (in Chinese). Chin J Soc Med. (2020) 37:465-467. doi:10.3969/j.issn.1673-5625.2020.05.004 | Management of mobile cabin hospitals   - Management of nosocomial infections |
| CNKI | 刘珺,张向阳,邓略,张晓丽,张莉莉,贺青,曹婧.信息化野战方舱医院在新冠肺炎疫情防控中的卫勤保障作用[J].中国数字医学,2020,15(09):12-14+33.  Liu J, Zhang XY, Deng L, Zhang XL, Zhang LL, He Q, et al. The Effect of Health Service Support of the Informationized Mobile Field Hospital in the Prevention and Control of COVID-19 (in Chinese). China Digital Med. (2020) 15:12-14+33. doi:10.3969/j.issn.1673-7571.2020.09.003 | Management of mobile cabin hospitals   - Information technology support |
| CNKI | 黄青,曾伟,蔡永辉.应对突发公共卫生事件的方舱医院运行管理标准化研究[J].中国标准化,2020(08):43-47.  Huang Q, Zeng W, Cai YH. Research on Operation and Management Standardization of Makeshift Hospital in Response to Public Health Emergency (in Chinese). China Stand. (2020) 8:43-47. doi: 10.3969/j.issn.1002-5944.2020.08.002 | Management of mobile cabin hospitals   - Organizational management |
| CNKI | 罗西贝,凌瑞杰,丁亚兴,王一颖.武汉江岸方舱医院医院感染预防与控制措施及效果评价[J].中国病毒病杂志,2020,10(04):284-288.  Luo XB, Ling RJ, Ding YX, Wang YY. Evaluation of measures for prevention and control of health care associated infections in Wuhan Jiang’an Shelter Hospital during the outbreak of coronavirus disease-19 (in Chinese). Chin J Viral Dis. (2020) 10:284-288. doi:10.16505/j.2095-0136.2020.0029 | Management of mobile cabin hospitals   - Management of nosocomial infections |
| CNKI | 傅新巧,孙晖,辛艳姣,孙扬,徐小兵,舒琴,张义丹,杨超,李迪.突发公共卫生事件应急管理中方舱医院医疗管理实践与思考——以江汉方舱医院为例[J].医学与社会,2020,33(05):86-89.  Fu XQ, Sun H, Xin YJ, Sun Y, Xu XB, Shu Q, et al. Practice and Thinking on Medical Management of Cabin Hospitals in Emergency Management of Public Health Emergency: Taking Jianghan Cabin Hospital as an Example (in Chinese). Med Soc. (2020) 33:86-89. doi:10.13723/j.yxysh.2020.05.018 | Management of mobile cabin hospitals   - Organizational management |
| CNKI | 张贝贝,李成伟,肖辉.新型冠状病毒肺炎疫情下方舱医院信息系统设计[J].中国数字医学,2020,15(05):11-13+57.  Zhang BB, Li CW, Xiao H. Design of the Information System of the Cabin Hospital Under COVID-19 (in Chinese). China Digital Med. (2020) 15:11-13+57. doi:10.3969/j.issn.1673-7571.2020.05.004 | Management of mobile cabin hospitals   - Information technology support |
| CNKI | 华小黎,辜明,罗立,曾芳,张玉,史琛.“零接触”信息化药学服务在医院防控新冠肺炎疫情中的应用[J].中国数字医学,2020,15(05):51-54.  Hua XL, Gu M, Luo L, Zeng F, Zhang Y, Shi C. The Application of "Zero Contact" Informationized Pharmaceutical Service in the Prevention and Control of COVID-19 in Hospitals (in Chinese). China Digital Med. (2020) 15:51-54. doi:10.3969/j.issn.1673-7571.2020.05.018 | Management of mobile cabin hospitals   - Information technology support |
| CNKI | 周君,杨勇,黄雷,陈康,张文超,熊伟.疫情期间“方舱医院”医药护协作医院感染防控模式实践与效果[J].中国药业,2020,29(09):40-43.  Zhou J, Yang Y, Huang L, Chen K, Zhang W, Xiong W. Practice and Effect of Physician – Pharmacist - Nurse Cooperation Mode in the Prevention and Control of Nosocomial Infection in Fangcang Hospitals (in Chinese). China Pharm. (2020) 29:40-43. doi:10.3969/j.issn.1006-4931.2020.09.011 | Management of mobile cabin hospitals   - Management of nosocomial infections |
| CNKI | 席新学,汪晖,毛靖,何细飞,刘美,余洪兴.方舱医院中新型冠状病毒肺炎患者的安全管理[J].中华护理杂志,2020,55(S1):53-55.  Xi XX, Wang H, Mao J, He XF, Liu M, Yu HX. Difficulties and coping strategies of safety management for inpatients with coronavirus disease 2019 at cabin hospital (in Chinese). Chin J Nurs. (2020) 55:53-55. doi:10.3761/j.issn.0254-1769.2020.S1.019 | Management of mobile cabin hospitals   - Management of nosocomial infections |
| CNKI | 周其源,洪瑛,宛超.方舱医院给排水设计要点[J].华中建筑,2020,38(04):123-125.  Zhou QY, Hong Y, Wan C. Key Points of the Water Supply and Drainage Design for Mobile Cabin Hospital (in Chinese). Huazhong Arch. (2020) 38:123-125. doi: 10.13942/j.cnki.hzjz.2020.04.030 | Preparedness of mobile cabin hospitals   - Conversion of public venues into hospital |
| CNKI | 陈顺达,汤杰,叶青,刘东.方舱医院无接触药品调剂模式实践[J].医药导报,2020,39(07):940-942.  Chen SD, Tang J, Ye Q, Liu D. Practice and Discussion of Non-contact Drug Dispensing Pattern in Square Cabin Hospital (in Chinese). Her Med. (2020) 39:940-942. doi:10.3870/j.issn.1004-0781.2020.07.011 | Management of mobile cabin hospitals   - Information technology support |
| CNKI | 苏玓,马黎,黄雷,陈康,王懿睿,张恩景,杨勇,孟敏.基于方舱医院药事管理对突发公共卫生事件药学服务体系建设的思考[J].中国药业,2020,29(07):16-18.  Su D, Ma L, Huang L, Chen K, Wang YR, Zhang EJ, et al. Construction of Pharmaceutical Care System for Public Health Emergencies Based on the Pharmaceutical Administration in Wuhan Fangcang Hospitals (in Chinese). China Pharm. (2020) 29:16-18. doi:10.3969/j.issn.1006-4931.2020.07.004 | Management of mobile cabin hospitals   - Information technology support |
| CNKI | 吴文娟,何丽华,刘波,王莉,雷撼,向准.新冠肺炎疫情期间方舱医院院内感染管理探讨[J].中华医院管理杂志,2020(04):320-321-322-323.  Wu WJ, He LH, Liu B, Wang L, Lei H, Xiang Z. Nosocomial infection control strategy in cabin hospitals during the epidemic of COVID-19 (in Chinese). Chin J Hosp Admin. (2020) 36:320-323. doi:10.3760/cma.j.cn112225-20200301-00418 | Management of mobile cabin hospitals   - Management of nosocomial infections |
| CNKI | 张义丹,丁宁,胡豫,孙晖,傅新巧,喻姣花,许栋,张明,张进祥.新冠肺炎疫情下武汉方舱医院运行管理模式及实践探析[J].中华医院管理杂志,2020(04):281-282-283-284-285.  Zhang YD, Ding N, Hu Y, Sun H, Fu XQ, Yu JH, et al. Exploration and analysis of the management mode of a cabin hospital during the outbreak of COVID-19 (in Chinese). Chin J Hosp Admin. (2020) 36:281-285. doi:10.3760/cma.j.cn112225-20200226-00320 | Management of mobile cabin hospitals   - Organizational management |
| CNKI | 姚刚,张晓祥,汪火明,李金,田坚,王梁.新冠肺炎疫情期间方舱医院信息化建设实践与思考[J].中华医院管理杂志,2020(04):334-335-336.  Yao G, Zhang XX, Wang HM, Li J, Tian J, Wang L. Practice and thinking of the informationized cabin hospitals during COVID-19 epidemic (in Chinese). Chin J Hosp Admin. (2020) 36:334-336. doi:10.3760/cma.j.cn112225-20200218-00200 | Management of mobile cabin hospitals   - Information technology support |
| CNKI | 梁浩,李宏军,李志勇,许远超,李宝山,刘岩,酒淼,张川,宫玮,龚维科.《工业建筑改造为方舱医院的建设运营技术指南（试行）》编制解读[J].建设科技,2020(06):27-30.  Liang H, Li HJ, Li ZY, Xu YC, Li BS, Liu Y, et al. Compilation Interpretation of “Technical Guidelines for Construction and Operation of the Industrial Buildings Renovated to Mobile Cabin Hospitals (Trial)” (in Chinese). Constr Sci Technol. (2020) 403:27-30. doi: 10.16116/j.cnki.jskj.2020.06.006 | Preparedness of mobile cabin hospitals   - Site selection - Conversion of public venues into hospital |
| CNKI | 龚卫静,周涛,徐晨枫,徐佳强,刘易慧,韩勇,曾芳,张玉.方舱医院线上药学服务模式的实践与探讨[J].中国医院药学杂志,2020,40(08):876-879.  Gong WJ, Zhou T, Xu CF, Xu JQ, Liu YH, Han Y, et al. The practice and discussion of online pharmaceutical service mode in mobile cabin hospital (in Chinese). Chin J Hosp Pharm. (2020) 40:876-879. doi:10.13286/j.1001-5213.2020.08.08 | Management of mobile cabin hospitals   - Information technology support |
| References | Sun S, Xie Z, Yu K, Jiang B, Zheng S, Pan X. COVID-19 and healthcare system in China: challenges and progression for a sustainable future. Global Health. (2021) 17:14. doi: 10.1186/s12992-021-00665-9 | COVID-19 and healthcare system |
| References | Kuppalli K, Gala P, Cherabuddi K, Kalantri SP, Mohanan M, Mukherjee B, et al. India's COVID-19 crisis: a call for international action. Lancet. (2021) 397:2132-2135. doi: 10.1016/s0140-6736(21)01121-1 | COVID-19 and healthcare system |
| References | The Indian Express. Dr Anthony S Fauci on India’s Covid Crisis: ‘Shut down the country for a few weeks…hang in there, take care of each other, we’ll get to a normal’ (2021). https://indianexpress.com/article/express-exclusive/indias-covid-crisis-anthony-s-fauci-coronavirus-death-7297380 [Accessed May 1, 2021]. | Effect and significance of mobile cabin hospital during the COVID-19 pandemic |
| References | Chen S, Zhang Z, Yang J, Wang J, Zhai X, Bärnighausen T, et al. Fangcang shelter hospitals: a novel concept for responding to public health emergencies. Lancet. (2020) 395:1305-1314. doi: 10.1016/s0140-6736(20)30744-3 | Effect and significance of mobile cabin hospital during the COVID-19 pandemic |
| References | Sun C, Wu Q, Zhang C. Managing patients with COVID-19 infections: a first-hand experience from the Wuhan Mobile Cabin Hospital. Br J Gen Pract. (2020) 70:229-230. doi: 10.3399/bjgp20X709529 | Effect and significance of mobile cabin hospital during the COVID-19 pandemic |
| References | Shi F, Wen H, Liu R, Bai J, Wang F, Mubarik S, et al. The comparison of epidemiological characteristics between confirmed and clinically diagnosed cases with COVID-19 during the early epidemic in Wuhan, China. Glob Health Res Policy. (2021) 6:18. doi: 10.1186/s41256-021-00200-8 | Clinical manifestations of COVID-19 |
| References | Wang KW, Gao J, Song XX, Huang J, Wang H, Wu XL, et al. Fangcang shelter hospitals are a One Health approach for responding to the COVID-19 outbreak in Wuhan, China. One Health. (2020) 10:100167. doi: 10.1016/j.onehlt.2020.100167 | Effect and significance of mobile cabin hospital during the COVID-19 pandemic |
| References | Zhu H, Wei L, Niu P. The novel coronavirus outbreak in Wuhan, China. Glob Health Res Policy. (2020) 5:6. doi: 10.1186/s41256-020-00135-6 | COVID-19 and healthcare system |
| References | Fang D, Pan S, Li Z, Yuan T, Jiang B, Gan D, et al. Large-scale public venues as medical emergency sites in disasters: lessons from COVID-19 and the use of Fangcang shelter hospitals in Wuhan, China. BMJ Glob Health. (2020) 5:e002815. doi: 10.1136/bmjgh-2020-002815 | Preparedness of mobile cabin hospitals   - Site selection - Conversion of public venues into hospital - Architectural layout |
| References | Li J, Yuan P, Heffernan J, Zheng T, Ogden N, Sander B, et al. Fangcang shelter hospitals during the COVID-19 epidemic, Wuhan, China. Bull World Health Organ. (2020) 98:830-841. doi: 10.2471/blt.20.258152 | Effect and significance of mobile cabin hospital during the COVID-19 pandemic |
| References | WHO. Prevention and control of outbreaks of seasonal influenza in long-term care facilities: A review of the evidence and best-practice guidance (2017). http://www.euro.who.int/__data/assets/pdf_file/0015/330225/LTCF-best-practice-guidance.pdf?ua=1 [Accessed September 4, 2017]. | Cohort nursing |
| References | Cepeda JA, Whitehouse T, Cooper B, Hails J, Jones K, Kwaku F, et al. Isolation of patients in single rooms or cohorts to reduce spread of MRSA in intensive-care units: prospective two-centre study. Lancet. (2005) 365: 295-304. doi: 10.1016/s0140-6736(05)17783-6 | Cohort nursing |
| References | Chen C, Zhao B. Makeshift hospitals for COVID-19 patients: where health-care workers and patients need sufficient ventilation for more protection. J Hosp Infect. (2020) 105:98-99. doi: 10.1016/j.jhin.2020.03.008 | Effect and significance of mobile cabin hospital during the COVID-19 pandemic |
| References | Chen Y, Zhou M, Hu L, Liu X, Zhuo L, Xie Q. Emergency reconstruction of large general hospital under the perspective of new COVID-19 prevention and control. Wien Klin Wochenschr. (2020) 132:677-684. doi: 10.1007/s00508-020-01695-w | Effect and significance of mobile cabin hospital during the COVID-19 pandemic |
| References | Wang J, Shen J, Ye D, Yan X, Zhang Y, Yang W, et al. Disinfection technology of hospital wastes and wastewater: Suggestions for disinfection strategy during coronavirus Disease 2019 (COVID-19) pandemic in China. Environ Pollut. (2020) 262:114665. doi: 10.1016/j.envpol.2020.114665 | Management of mobile cabin hospitals   - Management of nosocomial infections |
| References | Wang JQ, Liu XL, Duan HY, Chen XM, Qian L, Lyu XF, et al. Disinfection and Protective Measures for Makeshift Hospitals. Biomed Environ Sci. (2020) 33:940-942. doi: 10.3967/bes2020.129 | Management of mobile cabin hospitals   - Management of nosocomial infections |
| References | Hua X, Gu M, Zeng F, Hu H, Zhou T, Zhang Y, et al. Pharmacy administration and pharmaceutical care practice in a module hospital during the COVID-19 epidemic. J Am Pharm Assoc. (2020) 60:431-438.e1. doi: 10.1016/j.japh.2020.04.006 | Management of mobile cabin hospitals   - Information technology support |
| References | Zhang Y, Ding Q, Liu JB. Performance evaluation of emergency logistics capability for public health emergencies: perspective of COVID-19. Int J Logist Res App. (2021). doi: 10.1080/13675567.2021.1914566 | Management of mobile cabin hospitals   - Material supply |
| References | Cao Y, Shan J, Gong Z, Kuang J, Gao Y. Status and Challenges of Public Health Emergency Management in China Related to COVID-19. Front Public Health. (2020) 8:250. doi: 10.3389/fpubh.2020.00250 | Management of mobile cabin hospitals   - Material supply |
| References | Xing C, Zhang R. COVID-19 in China: Responses, Challenges and Implications for the Health System. Healthcare. (2021) 9:82. doi: 10.3390/healthcare9010082 | Management of mobile cabin hospitals   - Material supply |
| References | Li YR, Chandra Y, Kapucu N. Crisis Coordination and the Role of Social Media in Response to COVID-19 in Wuhan, China. Am Rev Public Adm. (2020) 50:698-705. doi: 10.1177/0275074020942105 | Management of mobile cabin hospitals   - Material supply |
| References | Zhang P. Study on the Experience of Public Health System Construction in China's COVID-19 Prevention. Front Public Health. (2021) 9:610824. doi: 10.3389/fpubh.2021.610824 | COVID-19 and healthcare system |

Supplementary Table 4. Basic information of the 16 mobile cabin hospitals in Wuhan, China. (The data are from studies of Fang D^[1]^ and Zhang Y^[2]^)

| Name | Original function | Construction time | Operation time | Number of beds | Number of admitted patients | Area (10000 m2) | Distance to the nearest neighbourhood (m) | Distance to the nearest designated hospital | Whether with a square | Whether along the main road |
| --- | --- | --- | --- | --- | --- | --- | --- | --- | --- | --- |
| Wuchang Mobile Cabin Hospital | Hongshan Sports Center | 2020.2.3 | 2.5– 3.10 | 784 | 1124 | 2 | 363 | 0.5 | Yes | Yes |
| Jianghan Mobile Cabin Hospital | Wuhan International Convention and Exhibition Center | 2020.2.3 | 2.5–3.9 | 1524 | 1848 | 12.7 | 162 | 1.6 | Yes | Yes |
| Dongxihu Mobile Cabin Hospital | Wuhan Living Room Culture Expo Center | 2020.2.3 | 2.7–3.8 | 1461 | 1760 | 12.5 | 959 | 0.8 | Yes | Yes |
| Hanyang Mobile Cabin Hospital | Wuhan International Expo Center | 2020.2.4 | 2.11–3.8 | 930 | 1028 | 18 | 883 | 2.9 | Yes | Yes |
| Qiaokou Mobile Cabin Hospital | Wuhan Stadium | 2020.2.4 | 2.11–3.1 | 200 | 330 | 1.45 | 133 | 1.3 | Yes | Yes |
| Huangpi Mobile Cabin Hospital | Huangpi District Gymnasium | 2020.2.4 | 2.11–3.7 | 200 | 223 | 0.74 | 550 | 2.1 | Yes | Yes |
| Jiangan Mobile Cabin Hospital | Tazihu Sports Center | 2020.2.4 | 2.12–3.8 | 900 | 1011 | 6.45 | 473 | 2.3 | Yes | Yes |
| Wuhan Economic Development Zone Mobile Cabin Hospital | Wuhan Sports Center | 2020.2.4 | 2.12–3.8 | 1000 | 1056 | 5.07 | 632 | 1 | Yes | Yes |
| Qingshan Mobile Cabin Hospital | Wuhan Iron and Steel Gymnasium | 2020.2.4 | 2.13–3.9 | 388 | 519 | 0.2 | 378 | 1.5 | Yes | Yes |
| Optical Valley Exhibition Center Mobile Cabin Hospital | Optical Valley Technology Exhibition Center | 2020.2.4 | 2.17–3.6 | 840 | 875 | 1 | 742 | 4.4 | Yes | Yes |
| Zhuankou Mobile Cabin Hospital | Zhuankou idle industrial plants | 2020.2.12 | 2.17–3.8 | 996 | 990 | 0.63 | 635 | 4.6 | Yes | No |
| East Lake Rihai Mobile Cabin Hospital | Original Rihai Industrial Park Factory | 2020.2.16 | 2.20-3.10 | 1300 | – | 5.4 | 319 | 5.3 | Yes | Yes |
| Jianghan Economic Development Zone Mobile Cabin Hospital | Industrial plants and warehouses | 2020.2.17 | 2.21–3.8 | 682 | 281 | 0.6 | 100 | 4.6 | Yes | Yes |
| Hanyang Sports School Mobile Cabin Hospital | Wuhan Sports School | 2020.2.19 | 2.22–3.8 | 1160 | 265 | 0.24 | 170 | 0.8 | Yes | Yes |
| Jiangxia Dahuashan Mobile Cabin Hospital | Dahuashan Outdoor Sports Center | 2020.2.4 | 2.14–3.10 | 420 | 564 | 7.95 | 577 | 3.1 | Yes | Yes |
| Provincial Party School Mobile Cabin Hospital | Provincial Party School Students Dormitory | 2020.2.7 | 2.19–3.1 | 932 | – | 0.2 | 100 | 4.8 | Yes | Yes |

References:

[1] Chen Y, Zhou M, Hu L, Liu X, Zhuo L, Xie Q. Emergency reconstruction of large general hospital under the perspective of new COVID-19 prevention and control. Wien Klin Wochenschr. (2020) 132:677-684. doi: 10.1007/s00508-020-01695-w

[2] Zhang Y, Shi L, Cao Y, Chen H, Wang X, Sun G. Wuhan mobile cabin hospital: A critical health policy at a critical time in China. Medicine. (2021) 100:e24077. doi: 10.1097/md.0000000000024077
